# Multi-scale modeling of the circadian modulation of learning and memory

**DOI:** 10.1371/journal.pone.0219915

**Published:** 2019-07-19

**Authors:** Shiju S, K. Sriram

**Affiliations:** Center for Computational Biology, Indraprastha Institute of Information Technology-Delhi, New Delhi, India; Georgia State University, UNITED STATES

## Abstract

We propose a multi-scale model to explain the time-of-day effects on learning and memory. We specifically model the circadian variation of hippocampus (HC) dependent long-term potentiation (LTP), depression (LTD), and the fear conditioning paradigm in amygdala. The model we built has both Goodwin type circadian gene regulatory network (GRN) and the conductance model of Morris-Lecar (ML) type to explain the spontaneous firing patterns (SFR) in suprachiasmatic nucleus (SCN). In the conductance model, we also include N-Methyl-D-aspartic acid receptor (NMDAR) to study the circadian dependent changes in LTP/LTD in hippocampus and include both NMDAR and *α*-amino-3-hydroxy-5-methyl-4-isoxazolepropionic acid receptor (AMPAR) dynamics to explain the circadian modulation of fear conditioning paradigm in memory acquisition, recall, and extinction as seen in amygdala. Our multi-scale model captures the essential dynamics seen in the experiments and strongly supports the circadian time-of-the-day effects on learning and memory.

## Introduction

Circadian rhythms are biological oscillations with a period close to 24h, which modulates different physiological and behavioral processes in all the living organisms. SCN is the master circadian oscillator in mammals, which consists of ≈ 20000 neurons, and exhibits endogenous rhythm in gene expression and generate action potentials with varying rates during day and night. The highly complex circadian GRN is regulated by intricate feedback and feedforward loops. The molecular clock consists of activators (*Clock* and *Bmal*1) that activates the repressors (*per* and *cry*), which in turn, after a delay, suppresses the activity of the activators and thereby inhibiting its own production. There are also many other major circadian genes and proteins that play a key role in the regulation of the functions of oscillator [1]. Electrical activity in the SCN neuron also shows daily variations that is important to synchronize the timing of the system throughout the body.

The role of circadian rhythms in modulating memory has been studied for a long time, yet molecular mechanisms and networks responsible for the modulation is not well understood. Clock genes that contribute to synaptic plasticity, and fear conditioning processes like acquisition, renewal, and extinction are partially known. Though its known that circadian rhythm is pervasive all through the system with SCN as a master clock, the regulation of peripheral slave circadian oscillators is far from understood. It is also equally important to consider the electrophysiological properties of SCN neurons controlled by ionic currents which in turns depends on the conductance of ionic channels to understand the cause of different firing patterns that occur during one circadian cycle. The mean value of conductance of SCN neurons was shown vary in a circadian manner [2]. However, the interactions among the GRN, conductance and the neurotransmitters in bringing about firing pattern variations are not clearly elucidated. For example, frequency of autonomously generated action potential in SCN is higher during the daytime than at night [3, 4], and how circadian dynamics modulates this firing patterns are not well characterized. Recently, Jones et al. [5] reported that *per1* plays an important role in phasing molecular gene expression and firing rate rhythm in SCN neuron. However, the relationship between the firing rate rhythm and the circadian molecular network is not fully understood.

Besides SCN, circadian rhythmicity have been identified in other brain regions that include amygdala and hippocampus [6–14]. Hippocampus and amygdala are the centers for learning, memory formation, and storage. Presently, memory is understood to be an electrically encoded representation of an event described by LTP and LTD. LTP/LTD refers to the strengthening/weakening of synaptic connection between the neurons and this dynamics is commonly referred to as synaptic plasticity. The measure of LTP (LTD) generally tracks either an increase (decrease) in the extra-cellular population excitatory postsynaptic potential (EPSP) or population spike (PS) [15]. EPSP makes postsynaptic neuron more likely to fire a spike (action potential), whereas, PS is the measure of the number of cells discharging spikes in response to stimulus [15]. Nabavi et al. [16] showed that associative memory can be activated by LTP and inactivated by LTD, which supported the hypothesis that memories are encoded by synaptic plasticity through LTP and LTD [17]. On the other hand, it is debated whether LTP/LTD is the actual phenomena responsible for memory [18]. The role of circadian rhythms in influencing LTP and LTD has been studied for a long time. Chaudhury et al. [19] found that LTP in the CA1 region of mice showed diurnal variation, where LTP during night is higher than day. Under constant dark (DD) condition, LTP shows higher magnitude during subjective night indicating the endogenous circadian rhythm modulation of the synaptic plasticity [19]. Endogenous circadian rhythm can also modulate different type of hippocampus specific memories and memory processes [20], and, SCN has intricate neural circuit connecting hippocampus that successfully mediates hippocampal activation [8, 14]. These experimental results showed that the circadian rhythm has significant influence on modulating LTP/LTD, but the exact molecular mechanism is not fully understood [6].

LTP and LTD are considered as the principal mechanism underlying learning and memory. Learning is the process of obtaining new information or remodel these existing characters. In associative learning process, a new response becomes associated with a specific event or stimulus. Classical fear conditioning is a form of associative learning in which the conditioned stimulus (CS) such as tone or context acquire the ability to evoke a fear response after the paired exposure of CS with the natural unconditioned stimulus (US) such as foot shock, and this associative learning task is an important technique for exploring the neurobiological basis of learning and memory. The opposite of learning, which is called extinction, is the repeated application of CS alone that results in a declined fear response [21]. Amygdala is responsible for controlling emotional memories and is also the region considered to study the fear conditioning paradigm and extinction [22] extensively. Savalli et al. [13] have shown that the aberrant circadian gene expression and loss of synchrony led to depression in the mouse model, while Chaudhury et al. [14] performed experiments to study the effect of circadian modulation of fear conditioned learning and memory. In particular, Chaudhury et al. [14] showed that during fear conditioning, acquisition is higher in the mice trained during the day than in the night. Recall ability for tone and context also varied as a function of time-of-the-day, and was found to be independent of the time of training. Moreover, mice trained in the night showed a greater degree of extinction than during the day. These results suggest that circadian rhythm modulates acquisition, recall, and extinction in the fear-conditioned mice for which the exact molecular mechanisms are not known.

Based on the above experimental evidences, two explanation about the influence of circadian rhythms on learning and memory are proposed: (i) circadian rhythms only modulate learning and memory, and (ii) clock is embedded in the system and it continuously perceives the time-of-the-day effects. Cain et al. [23] showed that learning and memory depends on the time-of-the-day that training had occurred. In their experiments, learning processes are preserved even when animals have lesioned SCN, indicating strongly that time-of-the-day information is implicitly encoded. They also speculated that the circadian rhythm may modulate the memory process independent of SCN. Also, since the time-scale of circadian rhythm dynamics is around 24 *h* and the learning, memory, and LTP/LTD processes take place at a much smaller time scales, ranging from millisecond to hours, they indicated that the circadian rhythms cannot modulate the learning and memory processes. Therefore, presently, in the absence of any molecular mechanism, it is not clear how a slow circadian process modulates the fast learning and memory processes. These findings suggest that no conclusive evidence can be arrived at to confirm or ignore the hypothesis that circadian rhythm modulates learning, memory, and LTP/LTD processes. However, based on the current newly obtained experimental evidence, we believe that circadian rhythm modulates cognitive functions in a phase-dependent manner [12, 14, 19, 24, 25].

To understand the influence of circadian dynamics on learning and memory, mathematical models can provide considerable insight into intricate working details of the mechanisms. While the mathematical models of circadian rhythms based on nonlinear ordinary differential equation (ODE) models of GRN’s has been popular and have been widely used to understand various dynamics of the system, conductance model of circadian rhythms are used to understand the electrophysiological properties [26–40]. Sim et al. [33] developed electrophysiological model of SCN, where they fit experimentally observed ionic current to Hodgkin-Huxley-type (HH) model. Later, by incorporating GRN of SCN, multi-scale models of SCN were developed and these models reproduced experimentally observed circadian variation of firing patterns [34–40]. Vasalou et al. [34] incorporated integrate and fire model (IF) and a 16 variable GRN [28] with calcium as a coupling agent. Diekman et al. [35] and Belle et al. [37] coupled Goodwin type GRN to HH model to explain the daily rhythm in firing patterns. Similarly, Dewoskin et al. [38] proposed an HH model coupled to a detailed mammalian circadian GRN model [29] to explain the firing pattern variations. Shiju et al. [36] performed multi-scale stochastic simulation, where they explained the role of noise and GRN in bringing about the circadian variation of SFR. On the other hand, many different types of models are proposed to capture the mechanism of synaptic plasticity, especially the models for LTP/LTD and spike time dependent plasticity (STDP) [41–45]. A detailed review of the models for LTP/LTD/STDP and their characteristics properties were discussed in [46]. Computational models for fear conditioning at amygdala were also previously developed [47–51], and explained the mechanism and dynamics behind learning, recall and the extinction. Presently, there are many circadian models of gene regulatory networks are available, but we construct a minimal model with only one clock protein *per2*/PER2 that plays a role in learning and memory.

Though these models are built and studied to understand specific properties, the interlink between circadian rhythms and synaptic plasticity, learning and memory are not considered. To the best of our knowledge, a multi-scale model that includes circadian GRN and electrophysiology coupled by neurotransmitters is not currently available to explain the mechanism of circadian variation of LTP/LTD, learning and memory. We have therefore in this paper built a coupled oscillator model to study three aspects of circadian modulation: (1) SFR in SCN, (ii) LTP/LTD in hippocampus and (iii) fear conditioning, recall and extinction at amygdala. We also demonstrate the importance of calcium dynamics, NMDAR, and AMPAR evoked ionic currents in the circadian modulation of learning and memory. We use experimental data taken from previously published results on C-57/6 or C-3H mice [5, 14, 19] to validate our model.

## 1 Materials and method

We provide in this section the details of models we built to understand the circadian modulation of SFR, LTP/LTD, and fear conditioning. To model the circadian modulation of SFR in SCN, we choose Goodwin type GRN model, and to capture the voltage and current changes we consider the modified version of Morris-Lecar model (ML) [52]. To model the circadian modulation of LTP/LTD in hippocampus, we consider two Goodwin type GRN models– one for SCN and the other one for hippocampus coupled via a yet unknown neurotransmitter. We also couple the GRN to the modified version of ML model to capture the electrophysiological dynamics in hippocampus. Also, we include NMDAR receptor dynamics to explain the occurrence of LTP/LTD and spike time dependent plasticity (STDP). To model the circadian modulation of fear conditioning, recall, and extinction in amygdala, we again consider two Goodwin type GRN model, one for the SCN and the other for amygdala, coupled via a neurotransmitter. We also consider a modified version of ML to capture NMDAR and AMPAR electrophysiological receptor dynamics. In all the three cases we explicitly include calcium dynamics that plays an important role in the circadian modulation of SFR in SCN, LTP/LTD in hippocampus, learning, and memory in amygdala. Details of the model are given in the subsequent part of this section.

### 1.1 Model for circadian modulation of firing pattern variation in SCN

To capture the firing pattern variations in SCN, we choose two models that have disparate time scales: (1) gene regulatory model of Goodwin type circadian oscillator to capture the dynamics of mRNA and proteins in hours and (2) the modified version of ML model to capture the firing patterns in SCN in milliseconds. Goodwin type oscillator [53] consists of dynamical variables *per* mRNA (*M*_*Ps*_), PER protein (*P*_1*s*_), and phosphorylated PER protein (*P*_1*Ps*_). It essentially describes the production of mRNA and protein, their degradation, and delayed-negative feedback of phosphorylated protein using Hills equation. These equations are highly nonlinear, which are given as:

$$\begin{array}{c} \frac{d}{dt}M_{Ps}=A_{s}\left(v_{s1}\frac{K_{As}^{n_{c}}}{K_{As}^{n_{c}}+P_{1s}^{n_{c}}}-M_{Ps}\right)+L \end{array}$$ (1)

$$\begin{array}{c} \frac{d}{dt}P_{1s}=A_{s}\left(M_{Ps}-P_{1s}\right) \end{array}$$ (2)

$$\begin{array}{c} \frac{d}{dt}P_{1Ps}=A_{s}\left(P_{1s}-P_{1Ps}\right) \end{array}$$ (3)

Where *A*_*s*_ is a scaling parameter and its value is 4.35*E* − 8 *ms*^−1^, *L* is the parameter responsible for light and its value under DD condition is 0. For LD cycle simulation, *L* is varied in a square wave manner with period 24 *h*, and we use *L* = 0.5*e* − 8 in the light phase, and *L* = 0 at the dark phase. Remaining parameter values are *v*_*s*1_ = 20 *nM*, *K*_*As*_ = 0.8 *nM*, *n*_*c*_ = 9. The model exhibits limit cycle oscillations with a period of 23.6 *h*, which is the typical free-running period of a mammalian circadian system [54].

To generate spontaneous firings, we use two-variable ML model, with *v* as the membrane voltage and *w* as the recovery variable that represents the fraction of *K*^+^ channel open at a given instant of time. The current-balance equation is given as:

$$\begin{array}{cc} C\frac{d}{dt}v & =I_{app}-I_{L}-I_{k}-I_{Ca}\\ & =I_{app}-g_{L}\left(v-v_{L}\right)-g_{K}w\left(v-v_{k}\right)-g_{Ca}m_{\infty }\left(v-v_{Ca}\right) \end{array}$$ (4)

$$\begin{array}{c} \frac{dw}{dt}=\lambda (w_{\infty }-w) \end{array}$$ (5)

$$\begin{array}{c} m_{\infty }=0.5(1+tanh\left(\frac{v-v_{1}}{v_{2}}\right)) \end{array}$$ (6)

$$\begin{array}{c} w_{\infty }=0.5(1+tanh\left(\frac{v-v_{3}}{v_{4}}\right)) \end{array}$$ (7)

$$\begin{array}{c} \lambda =\phi cosh(\frac{v-v_{3}}{2v_{4}}) \end{array}$$ (8)

Where *I*_*L*_, *I*_*K*_, and *I*_*Ca*_ are the leakage current, potassium current, and calcium current respectively. *w*_∞_ and *m*_∞_ are the open state probability functions of *K*^+^ and *Ca*^2+^ ions channel respectively. λ represents the voltage dependent time constant for *K*^+^ channel recovery variable. Parameter values of ML model are modified from the original model [52] to get experimentally observed firing patterns at different circadian phases and are given as *C* = 20 *pF*, *I*_*app*_ = 0 *mA*, *g*_*L*_ = 2 *nS*, *g*_*K*_ = 30 *nS*, *v*_*k*_ = −84*mV*, *v*_*Ca*_ = 90*mV*, *v*_*L*_ = −60*mV*, *v*_1_ = −1.2*mV*, *v*_2_ = 18*mV*, *v*_3_ = 12*mV*, *v*_4_ = 14.75*mV*, *ϕ* = 0.04.

To include the circadian modulation of SFR, we modify the conductance of calcium channel (*g*_*Ca*_) as a function of circadian variable *M*_*ps*_:

$$\begin{array}{c} g_{Ca}=g_{cabase}\frac{M_{ps}}{k_{ps}+M_{ps}} \end{array}$$ (9)

Where *g*_*cabase*_ = 6.37 *nS*, *k*_*ps*_ = 0.1 *nM*. We made this assumption based on the experimental observation that *Ca*^2+^ current in the SCN shows diurnal variation, and this current strongly contribute to the generation of spontaneous firing in membrane voltage [55, 56]. Previously, several computational models [34, 35, 37–39] have similarly incorporated circadian variation of calcium conductance based on experimental observation [55, 56].

### 1.2 Model for circadian modulation of LTP/LTD at hippocampus

**Fig 1:**
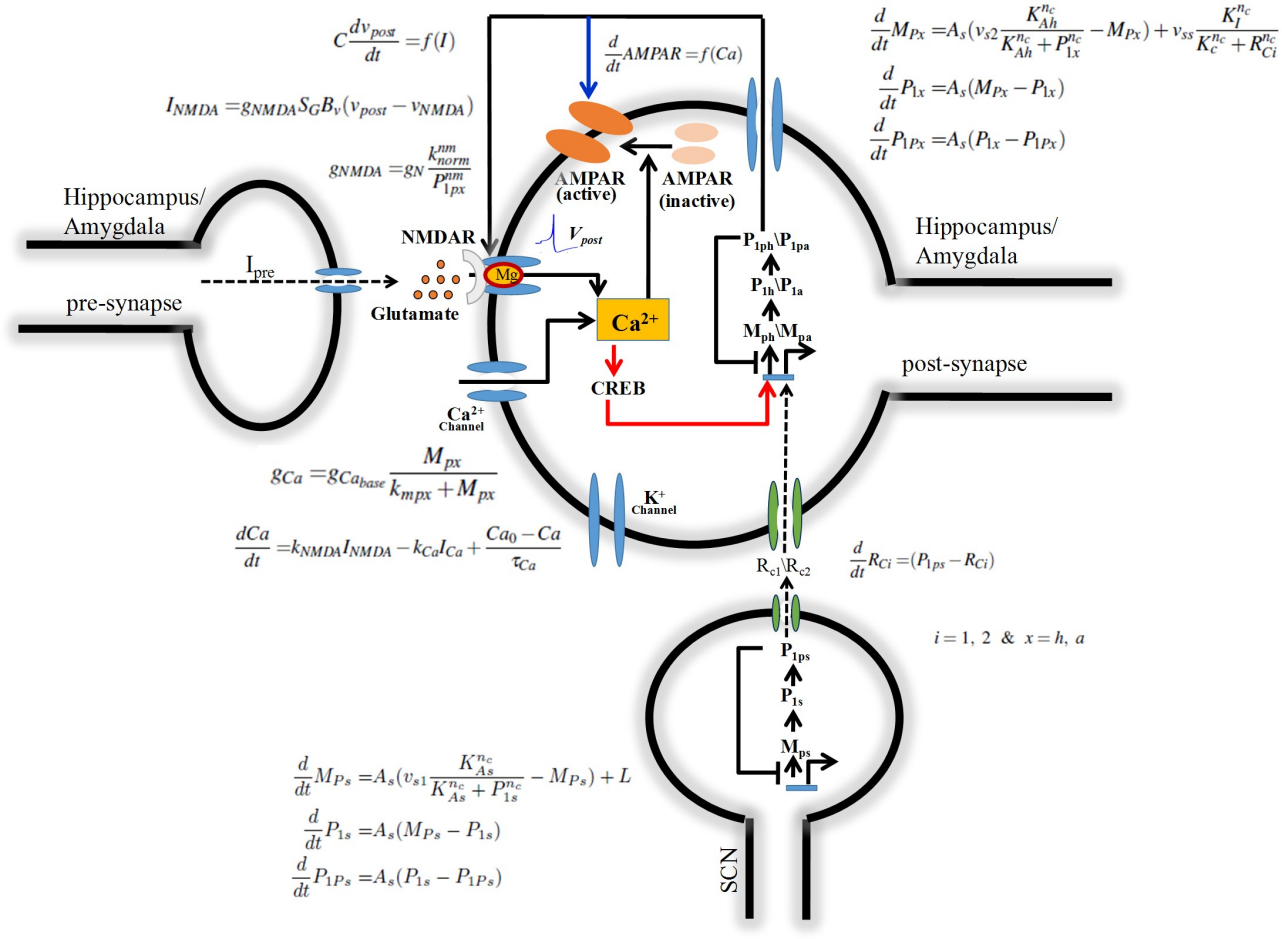
Multi-scale model for LTP/LTD, learning and memory. It is a unidirectionally coupled model with only SCN interacts hippocampus/amygdala and not vice-versa. Its a master-slave oscillator, with SCN acts as a master oscillator and hippocampus/amygdala as a slave oscillator. As mentioned earlier Goodwin type model is used to capture the SCN and hippocampus/amygdala circadian dynamics [53] which consists of 3 variables. These two Goodwin oscillators are coupled via an intermediate variable *R*_*C*1_/*R*_*C*2_. Hippocampus/amygdala model consists of pre and postsynaptic neurons. Due to presynaptic stimuli, glutamate is released and binds with NMDAR. These are permeable to calcium which is responsible for the insertion and removal of AMPAR. Conductance model of neurons are modified version of ML model. Rate equations and parameters are discuss in Materials and method section. Here red arrows indicate that the regulation is only present for the hippocampus model, while the blue arrows are only taken for amygdala.

To capture the circadian modulation of LTP/LTD at hippocampus, we consider the GRN Goodwin model for both SCN and hippocampus, the dynamics of NMDAR, AMPAR, Calcium, CREB, and the postsynaptic action potential. The GRN model of SCN and its parameter values are the same as described in the previous subsection. The GRN model of hippocampus consists of three dynamical variables, namely *per* mRNA (*M*_*Ph*_), PER protein (*P*_1*h*_), and phosphorylated PER protein (*P*_1*Ph*_). Previously, it was shown that when SCN is lesioned, there is a loss of *per2* oscillation [57] in hippocampus. We, therefore, assume that SCN drives the circadian oscillation in hippocampus that might function as a master-slave oscillator. As a result, we explain the circadian variation of LTP/LTD through coupled GRN model of SCN and hippocampus as shown in Fig 1. However, there is no information about how SCN oscillator drives the hippocampal circadian oscillator and maintains the antiphase relationship between them. Therefore, to capture this effect, we include an intermediate protein *R*_*C*1_ that couples SCN to hippocampus. It was reported that cAMP response element binding protein (CREB) dependent gene expression play a vital role in circadian system and synaptic palsticity [58, 59]. Therefore, we include the effect of calcium on hippocampal GRN by incorporating the CREB dependent transcription of *per* gene. The full GRN model equations are given as:

$$\begin{array}{cc} \frac{d}{dt}M_{Ph}= & A_{s}\left(v_{s2}\frac{K_{Ah}^{n_{c}}}{K_{Ah}^{n_{c}}+P_{1h}^{n_{c}}}-M_{Ph}\right)+v_{ss}\frac{R_{C1}^{n_{c}}}{K_{c}^{n_{c}}+R_{C1}^{n_{c}}}+\\ & K_{scr}S_{CREB}CREB \end{array}$$ (10)

$$\begin{array}{c} \frac{d}{dt}P_{1h}=A_{s}\left(M_{Ph}-P_{1h}\right) \end{array}$$ (11)

$$\begin{array}{c} \frac{d}{dt}P_{1Ph}=A_{s}\left(P_{1h}-P_{1Ph}\right) \end{array}$$ (12)

$$\begin{array}{c} \frac{d}{dt}R_{C1}=\left(P_{1ps}-R_{C1}\right) \end{array}$$ (13)

$$\begin{array}{c} \frac{d}{dt}S_{CREB}=a_{r}I_{pre}\left(1-S_{CREB}\right)-a_{d}S_{CREB} \end{array}$$ (14)

We estimate the parameters of hippocampus GRN model so that the oscillators in SCN and hippocampus are antiphase to each other as seen in the experiments [10, 60]. Parameter values are *A*_*s*_ = 4.35*e* − 5 *ms*^−1^, *v*_*s*2_ = 20 *nM*, *K*_*Ah*_ = 0.8 *nM*, *v*_*ss*_ = 1*e* − 3 *nMms*^−1^, *k*_*scr*_ = 1*e* − 4 *ms*^−1^, *K*_*c*_ = 5 *nM*, *n*_*c*_ = 9, *a*_*r*_ = 0.01 *pA*^−1^*ms*^−1^, *a*_*d*_ = 0.07 *ms*^−1^.

We do also consider pre and post-synaptic neurons in hippocampus where stimulus current from pre-synaptic neurons triggers the post-synaptic neurons. Postsynaptic neuron contains both AMPAR and NMDAR, and they are activated by binding of neurotransmitter glutamate (Glu) [61]. NMDAR has a high permeability for calcium ion, but it is blocked by magnesium (*Mg*^2+^) ion that prevents the calcium ion to enter through the receptor [62, 63]. Application of presynaptic stimulus current triggers the release of glutamate in postsynaptic neuron that binds to NMDAR and results in a very low permeability of ion that passes through the channel due to the *Mg*^2+^ blockade [64, 65]. However, when the postsynaptic neurons are sufficiently depolarized, *Mg*^2+^ blockade is removed from the NMDAR and results in a free flow of calcium into the postsynaptic neuron. Therefore NMDAR acts as a coincident detector since both presynaptic and postsynaptic events are required for the opening of ion channels [66]. The current associated with the ligand (Glutamate) and voltage-gated NMDAR is given as:

$$\begin{array}{c} I_{NMDA}=g_{NMDA}S_{G}B_{v}\left(v_{post}-v_{NMDA}\right) \end{array}$$ (15)

Where *v*_*NMDA*_ is the reverse potential of NMDAR current and based on the literature [42, 64, 67] value is taken as 0. The factor *B*_*v*_ is the *Mg*^2+^ blockade of NMDAR as described by [68], and here we modify the expression as:

$$\begin{array}{c} B_{v}=\frac{1}{1+0.005\left[Mg^{+}\right]e^{-0.2v_{postH}}} \end{array}$$ (16)

The dimensionless parameter [*Mg*^+^] has a value of 1. *g*_*NMDA*_ is the circadian time dependent conductance associated with the NMDAR, and it is given as:

$$\begin{array}{c} g_{NMDA}=g_{N}\frac{k_{norm}^{nm}}{P_{1ph}^{nm}} \end{array}$$ (17)

We made this assumption based on experimental observation that NMDAR functions exhibit circadian variations [9, 69–71]. Here *g*_*N*_ is the maximal conductance of NMDAR and its value is 12*nS*, *k*_*norm*_ has a unit of *nM* and its value is 1, and *n*_*m*_ = 2. The dimensionless gating variable *S*_*G*_ representing the fraction of glutamate binding site of NMDAR occupied at any given time. *S*_*G*_ = 0 in the absence of glutamate released from presynaptic neuron, and it has a non-zero value when *I*_*pre*_ is applied at presynaptic neuron, and its expression is given as:

$$\begin{array}{c} \frac{dS_{G}}{dt}=a_{r}I_{pre}\left(1-S_{G}\right)-a_{d}S_{G} \end{array}$$ (18)

Where *a*_*r*_ = 0.01 and *a*_*d*_ = 0.07. For postsynaptic membrane potential (*v*_*postH*_), we modified the ML model to include NMDAR evoked calcium current. Also, we modified the parameters in such a way that neuron produces an action potential when there is a postsynaptic current (*I*_*post*_) applied for a duration of 2*ms*.

$$\begin{array}{cc} C\frac{dv_{postH}}{dt}= & I_{post}-I_{L}-I_{k}-I_{Ca}-I_{NMDA}\\ = & I_{post}-g_{L}\left(v_{postH}-v_{L}\right)-g_{k}w\left(v_{postH}-v_{k}\right)-\\ & g_{Ca}m_{\infty }\left(v_{post}-v_{Ca}\right)-I_{NMDA} \end{array}$$ (19)

$$\begin{array}{c} \frac{dw}{dt}=\lambda (w_{\infty }-w) \end{array}$$ (20)

$$\begin{array}{c} m_{\infty }=0.5(1+tanh\left(\frac{v_{postH}-v_{1}}{v_{2}}\right)) \end{array}$$ (21)

$$\begin{array}{c} w_{\infty }=0.5(1+tanh\left(\frac{v_{postH}-v_{3}}{v_{4}}\right)) \end{array}$$ (22)

$$\begin{array}{c} \lambda =\phi cosh(\frac{v_{postH}-v_{3}}{2v_{4}}) \end{array}$$ (23)

For the numerical simulation we have *C* = 20*μF*/*cm*^2^, *g*_*L*_ = 2*μS*/*cm*^2^, *g*_*K*_ = 8*μS*/*cm*^2^, *v*_*k*_ = −84*mV*, *v*_*Ca*_ = 120*mV*, *v*_*L*_ = −60*mV*, *v*_1_ = −12*mV*, *v*_2_ = 18*mV*, *v*_3_ = 2*mV*, *v*_4_ = 30*mV*, *ϕ* = 0.08. As discussed in the previous section, here also we incorporate the circadian rhythmicity in calcium dynamics by making the conductance of calcium channel dependent on hippocampus GRN variable *P*_1*ph*_:

$$\begin{array}{c} g_{Ca}=g_{Ca-base}\frac{P_{1ph}}{k_{P1ph}+P_{1ph}} \end{array}$$ (24)

Where *g*_*cabase*_ = 4.35 *nS*/*cm*^2^, *k*_*P*1*ph*_ = 0.1 *nM*. It has been shown that NMDAR are the significant source of Calcium and it is vital for LTP/LTD due to pre-post synaptic activity [72, 73]. Therefore, in our model calcium enters the post synaptic neuron through NMDAR and voltage gated calcium channel. The dynamics of calcium concentration is determined by the following equation:

$$\begin{array}{c} \frac{dCa}{dt}=k_{NMDA}I_{NMDA}-k_{Ca}I_{Ca}+\frac{Ca_{0}-Ca}{\tau_{Ca}} \end{array}$$ (25)

Where, *Ca*_0_ = 500*nM*, *k*_*INMDA*_ = 1, *k*_*Ca*_ = 1, *τ*_*Ca*_ = 10*ms*.

The main procedure to evoke LTP/LTD involves an increase/decrease in the excitatory post-synaptic potential (EPSP) or the number of AMPAR within the postsynaptic membrane. EPSP defined as the potential due to excitatory postsynaptic current (EPSC), the current observed in response to the triggering of glutamate neuro transmitter [34]. In our model, calcium ion (*Ca*^2+^) is the major input for synaptic plasticity. Therefore, current due to NMDAR (*I*_*NMDAR*_) is treated as EPSC, and the voltage contributed by EPSC in postsynaptic potential (*v*_*postH*_) is treated as EPSP. Thus dynamics of EPSP is therefore given as:

$$\begin{array}{c} \frac{d}{dt}EPSP=I_{NMDA}/C \end{array}$$ (26)

Previously, in a modeling study, Pi and Lisman defined EPSP as proportional to the number of AMPAR on the synaptic membrane [41], where they showed that at the moment of synaptic activation EPSP changed from one stable steady state to another. Similarly, in our model, EPSP defined in Eq (26) is used to measure LTP/LTD, and this is qualitatively represent the LTP/LTD given in the literature [41, 74, 75].

*Ca*^2+^ is responsible for the insertion and removal of the AMPAR receptors in the neuronal membrane [73, 76], therefore we define the dynamics of AMPAR as a function of *Ca*^2+^, which is given as:

$$\begin{array}{cc} \frac{d}{dt}AMPAR= & (a_{r1}\left(Ca-Ca_{1}\right)\left(A_{m}-AMPAR\right)-\\ & a_{d1}AMPAR\left(Ca-Ca_{1}\right))S_{AMPA} \end{array}$$ (27)

$$\begin{array}{c} \frac{d}{dt}S_{AMPA}=a_{rs}I_{post}\left(1-S_{AMPA}-a_{ds}S_{AMPA}\right) \end{array}$$ (28)

Where, *a*_*r*1_ = 1*e* − 7, *A*_*m*_ = 1*e*4, *a*_*d*1_ = 1*e* − 5, *a*_*rs*_ = 0.01, *a*_*ds*_ = 0.007, *Ca*_1_ = 509.5.

### 1.3 Model for circadian modulation of learning and memory at amygdala

It has been shown that *per* genes in SCN and amygdala peaks during day time [13] and are in phase with each other. We, therefore explain the circadian modulation of learning and memory through a coupled model of SCN and amygdala. We took the same GRN model of SCN as described in the previous subsection and the GRN model of amygdala again consists of three dynamical variables, namely *per* mRNA (*M*_*pa*_), PER protein (*P*_1*a*_), phosphorylated PER protein (*P*_1*pa*_) and the intermediate protein *R*_*C*2_ which is responsible for coupling SCN to amygdala. The model equations are:

$$\begin{array}{c} \frac{d}{dt}M_{pa}=A_{s}\left(v_{s2}\frac{K_{Ah}^{n_{c}}}{K_{Ah}^{n_{c}}+P_{1a}^{n_{c}}}-M_{pa}\right)+v_{ss}\frac{K_{I}^{n_{c}}}{K_{c}^{n_{c}}+R_{C2}^{n_{c}}} \end{array}$$ (29)

$$\begin{array}{c} \frac{d}{dt}P_{1a}=A_{s}\left(M_{pa}-P_{1a}\right) \end{array}$$ (30)

$$\begin{array}{c} \frac{d}{dt}P_{1pa}=A_{s}\left(P_{1a}-P_{1pa}\right) \end{array}$$ (31)

$$\begin{array}{c} \frac{d}{dt}R_{C2}=\left(P_{1ps}-R_{C2}\right) \end{array}$$ (32)

Parameter values are *A*_*s*_ = 4.35*e* − 5 *ms*^−1^, *v*_*s*2_ = 20 *nM*, *K*_*Ah*_ = 0.8 *nM*, *v*_*ss*_ = 1*e* − 3 *nMms*^−1^, *K*_*c*_ = 5 *nM*, *n*_*c*_ = 9.

For postsynaptic membrane potential at amygdala (*v*_*postA*_), we modify the ML model to include glutamate controlled NMDAR and AMPAR evoked ionic currents (*I*_*NMDA*_, *I*_*AMPAR*_). Also, we modify the parameters in such a way that neurons produce an action potential when there is postsynaptic current (*I*_*post*_) applied for a duration of 2 *ms*.

$$\begin{array}{cc} C\frac{dv_{postA}}{dt}= & I_{post}-I_{L}-I_{k}-I_{Ca}-I_{NMDA}-I_{AMPAR}\\ = & I_{post}-g_{L}\left(v_{postA}-v_{L}\right)-g_{k}w\left(v_{postA}-v_{k}\right)-\\ & g_{Ca}m_{\infty }\left(v_{postA}-v_{Ca}\right)-I_{NMDA}-I_{AMPAR} \end{array}$$ (33)

$$\begin{array}{c} I_{AMPAR}=g_{AMPAR}S_{G}\left(v_{postA}-v_{AMPAR}\right) \end{array}$$ (34)

For the numerical simulation we have *C* = 20*μF*/*cm*^2^, *g*_*L*_ = 2*μS*/*cm*^2^, *g*_*K*_ = 8*μS*/*cm*^2^, *v*_*k*_ = −84*mV*, *v*_*Ca*_ = 120*mV*, *v*_*L*_ = −60*mV*. Here *v*_*AMPAR*_ is the reverse potential of AMPAR current and its value is taken as zero [42, 67].

It was reported that AMPAR is transported into and out of synapse during learned responses and it strengthens or weakens the synaptic function [77]. Rumpel et al. reported that at amygdala, fear conditioning pushes AMPAR into the synapse of post-synaptic neuron and hence mediate the encoding of memories [78]. Clem and Huganir found that extinction induced erasure of memory at amygdala was due to the synaptic removal of AMPAR [79]. Therefore, in our model, we assume that AMPAR will be recruit to synaptic membrane during acquisition and removed during extinction. We include the dynamics of addition and removal of AMPAR in the synapse as a function of both calcium and circadian variables:

$$\begin{array}{cc} \frac{d}{dt}AMPAR= & A_{MS}((a_{r1}\left(Ca-Ca_{1}\right)\left(A_{m}-AMPAR\right)-\\ & a_{d1}AMPAR\left(Ca-Ca_{1}\right))S_{AMPA})+\\ & P_{1pa}^{s_{s}}a_{remov}\left(\frac{I_{pre}}{1+I_{pre}}\right)\left(1-AMPAR\right) \end{array}$$ (35)

$$\begin{array}{c} A_{m}=A_{MS}A_{mx} \end{array}$$ (36)

$$\begin{array}{c} A_{MS}=\frac{k_{am1}^{q_{q}}}{P_{1pa}^{q_{q}}} \end{array}$$ (37)

Where *a*_*r*1_ = 1*e* − 7 *nM*^−1^*ms*^−1^, *A*_*m*_ = 1*e*4, *a*_*d*1_ = 1*e* − 5 *nM*^−1^*ms*^−1^, *Ca*_1_ = 509.5 *nM*, *A*_*mx*_ = 2500, *k*_*am*1_ = 1.3 *nM*, *q*_*q*_ = 2, *s*_*s*_ = 2.

It was reported that AMPAR levels are high during wake and low during sleep [80], which indicates that AMPAR functioning is also circadian dependent. Therefore we modeled the cumulative AMPAR conductance of postsynaptic neuron (*g*_*AMPAR*_) as a function of circadian variable *P*_1*pa*_, and it is given as:

$$\begin{array}{c} g_{AMPAR}=g_{AM}\frac{k_{am1}^{r_{r}}}{P_{1pa}^{r_{r}}}AMPAR \end{array}$$ (38)

Where *g*_*AM*_ = 0.2 *nS*, *k*_*am*1_ = 1.3 *nM*, *r*_*r*_ = 3. We consider AMPAR conductance, *g*_*AMPAR*_, to compare our model output with the experimental data and for model validation. Remaining model equations are the same as that discussed in the previous sections; equations and parameter values are given in that Table 1.

**Table 1:** Model equations and parameters for amygdala.

Equations	Parameters
*I*_*NMDA*_ = *g*_*NMDA*_*S*_*G*_*B*_*v*_(*v*_*postA*_ − *v*_*NMDA*_)	
$g_{NMDA}=g_{N}\frac{k_{norm}^{n_{m}}}{P_{1pa}^{n_{m}}}$	*v*_*NMDA*_ = 0 *mV*, *g*_*N*_ = 12 *nS*, *k*_*norm*_ = 1 *nM*, *n*_*m*_ = 2
$B_{v}=\frac{1}{1+0.005\left[Mg^{+}\right]e^{-0.2v_{postA}}}$	*Mg*^+^ = 1
$\frac{d}{dt}S_{G}=a_{r}I_{pre}\left(1-S_{G}\right)-a_{d}S_{G}$	*a*_*r*_ = 0.01 *pA*^−1^*ms*^−1^, *a*_*d*_ = 0.07 *ms*^−1^
$\frac{dw}{dt}=\lambda \left(w_{\infty }-w\right)$	
$m_{\infty }=0.5\left(1+tanh\left(\frac{v_{postA}-v_{1}}{v_{2}}\right)\right)$	*v*_1_ = −1.2*mV* *v*_2_ = 18*mV*, *v*_3_ = 2*mV*, *v*_4_ = 30*mV*, *ϕ* = 0.08
$w_{\infty }=0.5\left(1+tanh\left(\frac{v_{postA}-v_{3}}{v_{4}}\right)\right)$	
$\lambda =\phi cosh(\frac{v_{postA}-v_{3}}{2v_{4}})$	
$g_{Ca}=g_{cabase}\frac{M_{pa}}{k_{pa}+M_{pa}}$	*g*_*cabase*_ = 5.3 *nS*, *k*_*pa*_ = 0.01 *nM*
$\frac{d}{dt}Ca=k_{NMDA}I_{NMDA}-k_{Ca}I_{Ca}+\frac{Ca_{0}-Ca}{\tau_{Ca}}$	*Ca*_0_ = 500*nM*, *k*_*INMDA*_ = 10, *k*_*Ca*_ = 1, *τ*_*Ca*_ = 10*ms*
$\frac{d}{dt}EPSP=I_{NMDA}/C$	
$\frac{d}{dt}S_{AMPA}=a_{rs}I_{post}\left(1-S_{AMPA}\right)-a_{ds}S_{AMPA}$	*a*_*rs*_ = 0.01 *pA*^−1^*ms*^−1^, *a*_*ds*_ = 7*e* − 4 *ms*^−1^

### 1.4 Numerical simulation

There are two time scales in the model; circadian gene regulatory model with a slow time scale of 24h, and the conductance model with a time scale of milli-second (*ms*). We first convert all the circadian dynamics from hours to *ms* time scale by scaling the GRN model (by parameter *A*_*s*_ in all the GRN equations given in the supporting information). This involves integrating the equations for a long time to get the desired dynamics and leads to a generation of huge amount of data and long time to complete the simulation. Therefore, instead of performing numerical simulations of SFR, LTP/LTD, acquisition, recall and extinction simulation continuously for 1 circadian cycle, we evolve the equations by integrating the model with two different time steps; one long time step to simulate slow varying circadian dynamics and another with small time step to simulate the conductance model only for the desired circadian phases to get the corresponding voltage dynamics. Initially, we run the simulation with a long time step (*δt*1 = 10000*ms*, (0.0028*h*)) till we reach the desired circadian phase *t*_*des*_ at which we calculate the voltage dynamics. Once we reach *t* = *t*_*des*_, we reduce the time step to *δt*2 = 0.01*ms* and run the simulation till the desired time duration (*t*_*dur*_). Reducing *δt*1 (long time steps) from 10000*ms* to 1000*ms* and *δt*2 (short) from 0.01*ms* to 0.001*ms* yields the same result in our simulation. The summary of the procedure for solving multi-scale coupled oscillatory model is given as Algorithm-1. We use Xppaut [81] for numerical simulation and the program files are printed in the supplementary (S1 Program).

**Algorithm-1**: Solve the multi-scale coupled model at desired circadian time to get SFR, LTP, LTD, acquisition, recall, and extinction

**Input**: multi-scale coupled model

**Input**: step size (*δt*), *δt*_1_, *δt*_2_ (*δt*_1_ ≫ *δt*_2_)

**Input**: desired circadian time, *t*_*des*_

**Input**: time duration for calculation (*t*_*dur*_)

**Input**: presynaptic current, *I*_*pre*_

**Input**: postsynaptic current, *I*_*post*_

**Input**: time at which *I*_*pre*_ applied, *t*_*pre*_

**Input**: time at which *I*_*post*_ applied, *t*_*post*_

**If** *t* ≤ *t*_*des*_

*δt* = *δt*_1_

$\begin{array}{c} I_{post}=0\\ I_{pre}=0 \end{array}\}\begin{array}{c} \begin{array}{c} \text{for}\;\text{LTP},\;\text{LTD},\;\text{acquisition},\;\text{recall},\;\text{extinction} \end{array} \end{array}$

**else if** *t* > *t*_*des*_ & *t* < (*t*_*des*_ + *t*_*dur*_)

$\begin{array}{l} \delta t=\delta t_{2}\\ I_{post}>0\;for\;t_{post}<t>(t_{post}+2ms),\\ \begin{array}{ll} I_{pre}>0\;for\;t_{pre}<t>(t_{pre}+2ms)\; & \begin{array}{l} \text{for}\;\text{recall},\;\text{extinction} \end{array} \end{array} \end{array}\}\begin{array}{c} \begin{array}{c} \text{for}\;\text{LTP},\;\text{LTD},\;\text{acquisition} \end{array} \end{array}$

**end if**

$\begin{array}{l} v^{\prime }=f\left(I\right),\;\text{for}\;\text{SFR}\\ g_{AMPAR}=f(I_{pre},I_{post}) \end{array}\}\begin{array}{c} \begin{array}{c} \text{for}\;\text{LTP},\;\text{LTD},\;\text{acquisition},\;\text{recall},\;\text{extinction} \end{array} \end{array}$

## 2 Results

In this section we provide the results of the numerical simulation of the models for the circadian modulation of SFR at SCN, LTP/LTD including STDP at hippocampus, and the fear conditioning paradigm at amygdala. We also compare all the in-silico results with the experiments that are available.

### 2.1 Circadian modulation of SFR at SCN

**Fig 2:**
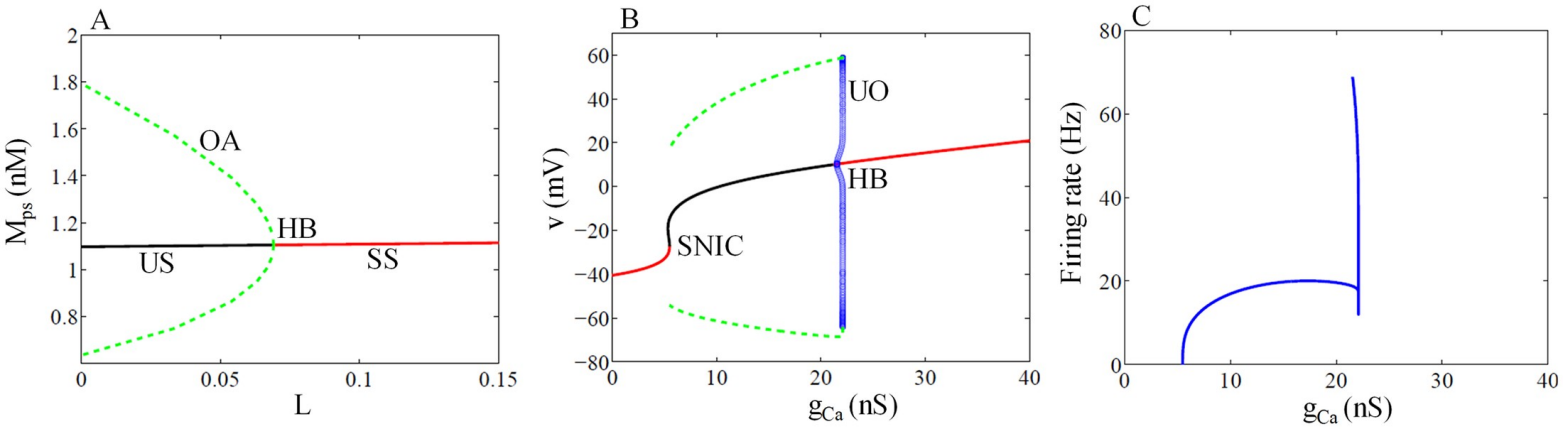
Bifurcation diagram for Goodwin and ML model. (A) Codimension-1 bifurcation diagram of GRN with L as the bifurcation parameter. At lower values of L, the system shows sustained oscillation. Broken green lines are the amplitude of the oscillation (OA), and black lines are the unstable steady state (US). As light intensity increases, sustained oscillation disappears via supercritical Hopf bifurcation (HB), and the system enters the stable steady state (red lines, SS). (B) Codimension-1 bifurcation diagram of modified ML model with *g*_*Ca*_ as bifurcation parameter. For lower values of *g*_*Ca*_, the system is in stable steady states (red line). As *g*_*Ca*_ increases stable steady state and unstable steady state merge each other and oscillations arise via SNIC bifurcation. Stable oscillations disappeared via subcritical Hopf bifurcation (HB) at *g*_*Ca*_ = 21.5 *nS*. Blue circles are unstable oscillation amplitude (UO). (C) Firing rate as a function of *g*_*Ca*_. When *g*_*Ca*_ = 5.49 *nS*, ML model starts firing with a frequency less than 0.5 Hz, when *g*_*Ca*_ = 6.35 *nS*, its firing rate increased to 10 Hz, and when *g*_*Ca*_ > 6.35 *nS*, firing rate become greater than 10Hz. Here we take $g_{Ca}=g_{Ca_{base}}$, the rest of the parameter values are the same as described in materials and method section. Xppaut [81] was used for simulating the bifurcation diagram.

Codimension-1 bifurcation diagram of the GRN model is shown in Fig 2A, with light L as the bifurcation parameter. For lower values of L, the model exhibit sustained oscillations, and when increased further, the system enters into a stable steady state (red line) via Hopf bifurcation, where the unstable steady state (black line) is surrounded by the stable limit cycle. We also similarly construct the codimension-1 bifurcation diagram for modified ML model with *g*_*Ca*_ as the bifurcation parameter (Fig 2B). For small values of *g*_*Ca*_, the system is in stable steady state (Fig 2B red line) and as *g*_*Ca*_ increases stable steady state is approached by an unstable stable steady state (black line), they merge each other, and oscillations are arise (green broken line) via saddle-node of infinite period/on invariant cycle (SNIC) bifurcation [82, 83]. Period of oscillation near SNIC is very large (frequency is small, Fig 2C). As *g*_*Ca*_ increases, period of oscillation decreases greatly (frequency increases, Fig 2C). Further increase in *g*_*Ca*_ leads to loss of periodic orbits through subcritical Hopf bifurcation (HB) and the system enters the stable steady state. We use Xppaut [81] to simulate all the bifurcation diagrams.

**Fig 3:**
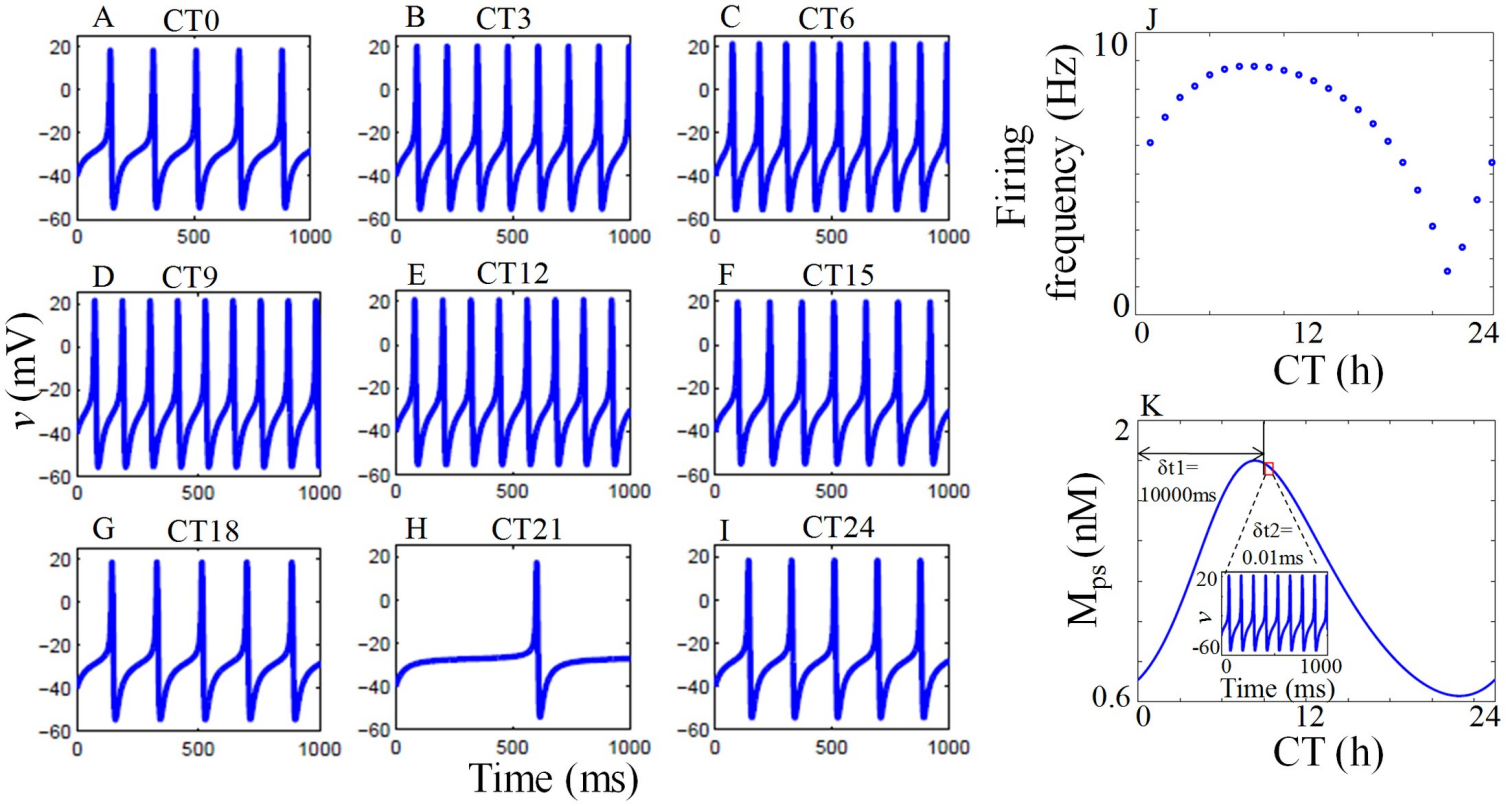
Circadian variation of firing frequency. (A-I) Time series of membrane voltage at different circadian phases. The number of spikes generated during the middle of subjective day (CT6) is higher in comparison to the middle of subjective night (CT18). A minimum number of spike is also observed during subjective night (CT21). (J) SFR as a function of circadian time. Firing rates show circadian variation that peaks during the day and reaches minimum value during the night, and this is in good agreement with the experimental results [5]. (K) Numerical simulation for SFR as described in the Algorithm-1. Here desired circadian time is CT9. From CT0 to CT9, the step size is 1000*ms*, and at CT9, the step size is 0.01*ms* for 1000*ms*, and the corresponding firing patterns obtained is used to calculate the firing frequency at CT9.

We choose the parameter *g*_*cabase*_ = 6.37 *nS* for the conductance model, and when coupled with GRN, it produces firing pattern in the frequency range from 0.5 Hz to 8.5 Hz as observed experimentally in SCN [5]. To simulate the firing patterns at different circadian phases, we couple the mRNA (*M*_*Ps*_) of GRN model to the conductance (*g*_*Ca*_) of ML model that modulates calcium conductance at different phases. The coupling term is added on the similar lines as in [34]. In the simulation, peaking of *M*_*Ps*_ is taken as CT8, which is the subjective day. We capture the firing rate that varies over 1 circadian cycle. We show in Fig 3A–3I, the modulation of firing rate by GRN for 1s at different circadian phases in one cycle. For example, the number of spikes at CT18 (night) is less compared to CT6 (day), which agrees with the experimental findings of [5] that SFR is higher during the day than the night. This important result clearly indicates that the circadian rhythm regulates the firing patterns. SFR as a function of circadian time is shown in Fig 3J, where firing rates show circadian variation that peaks during the day and reach minimum during the night. This is in good agreement with the experimental results [5]. Fig 3K shows the numerical simulation for SFR as described in Algorithm-1. Here, the desired circadian time is CT9. From CT0 to CT9 a step size of 1000*ms* is taken, and at CT9, the step size is reduced to 0.01*ms* for 1000*ms* to get the firing patterns from which the firing frequency at CT9 is calculated.

### 2.2 Circadian modulation of LTP/LTD at hippocampus

#### 2.2.1 Spike time dependent plasticity (STDP) without GRN

**Fig 4:**
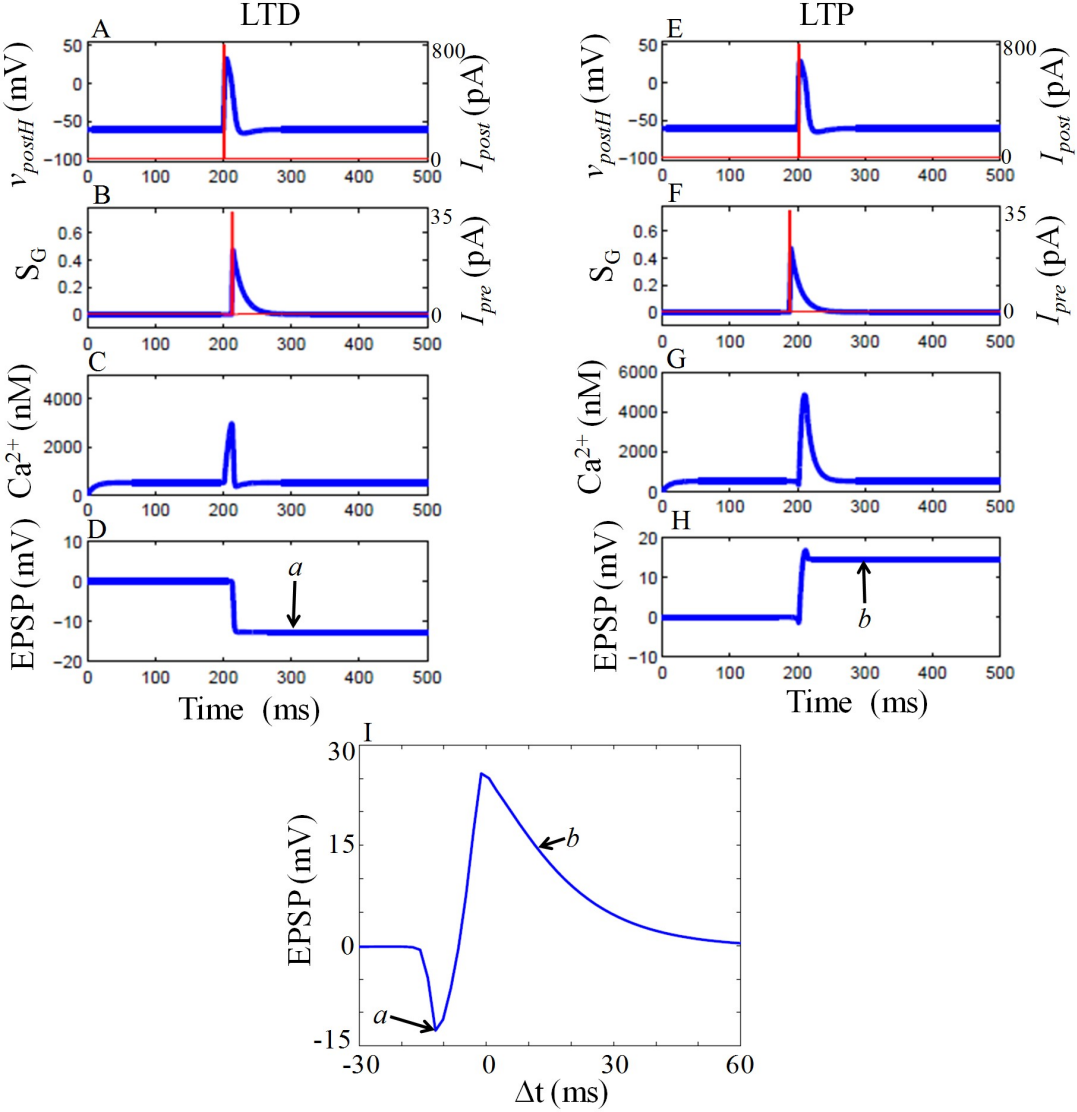
Time series of variables for STDP without GRN. In simulation, we apply *I*_*post*_ at *t* = 200*ms* (A, E, red line), which induce a spike at post-synaptic neuron (A, E, blue line). (B) When Δ*t* = −12*ms*, *I*_*pre*_ is applied at *t* = 212*ms* (red line). Glutamate is released, and only a fraction of glutamate binds to NMDAR (blue line). (C) Dynamics of the *Ca*^2+^. (D) Moderate increase of *Ca*^2+^ level induce a negative change in the EPSP, which is the measure of LTD. (F) When Δ*t* = + 12*ms*, *I*_*pre*_ applied at *t* = 188*ms* (red line), which trigger glutamate release and fraction of glutamate that binds on NMDAR (blue line). (G) Dynamics of *Ca*^2+^. In comparison to previous case in (C), increment of *Ca*^2+^ level is higher that results in a positive change in the EPSP (H), a measure of LTP. (I) *EPSP* as a function of Δ*t*. LTP and LTD occur when Δ*t* is positive or negative respectively. If the temporal difference between *I*_*pre*_ and *I*_*post*_ is large, then no LTP/LTD happens. EPSP value obtained at *t* = 300*ms* (*a*, *b*) is used to construct STDP curve. The magnitude of the *I*_*pre*_ and *I*_*post*_ are 35 pA and 800 pA respectively, and the duration of the current is 2*ms*. Here we take *g*_*NMDA*_ = *g*_*N*_ and *g*_*Ca*_ = *g*_*cabase*_.

In this section, we explain the pre and post-synaptic activity-dependent plasticity, STDP, through our model. STDP links the time difference between pre and postsynaptic spikes (Δ*t* = *t*_*post*_ − *t*_*pre*_) to synaptic changes (*EPSP*). If the presynaptic stimuli (*I*_*pre*_) applied at *t*_*pre*_ and postsynaptic spike generated by the application of current (*I*_*post*_) at *t*_*post*_, it evokes NMDAR dependent calcium dynamics and induces synaptic plasticity. This is shown in Fig 4. When Δ*t* is negative, *I*_*pre*_ is applied after *I*_*post*_ (Fig 4A and 4B). *I*_*pre*_ induces glutamate release at pre-synaptic neuron and *I*_*post*_ induces action potential at post-synaptic neuron(Fig 4A). Glutamate binds to NMDAR, and the percentage of glutamate that binds to NMDAR shown in Fig 4B. This allows NMDAR current to flow to elevate *Ca*^2+^ to a moderate level (Fig 4C) and as a result, induces a negative change in the *EPSP* (Fig 4D). Similarly, when Δ*t* is positive, presynaptic spikes arrive before postsynaptic spikes (Fig 4E and 4F). It is clear that the percentage of glutamate starts to bind to the NMDAR (Fig 4F) after the postsynaptic spike arrives and induces a higher level of calcium (Fig 4G) that in turn cause a positive change in the *EPSP* (Fig 4H). The overall change in the *EPSP* as a function of Δ*t* is shown in Fig 4I. Pre-synaptic spiking happens before or after the postsynaptic spiking results in a maximal EPSP change, while no EPSP change take place if the time difference between them is larger.

#### 2.2.2 Circadian variation of LTP/LTD under DD condition

**Fig 5:**
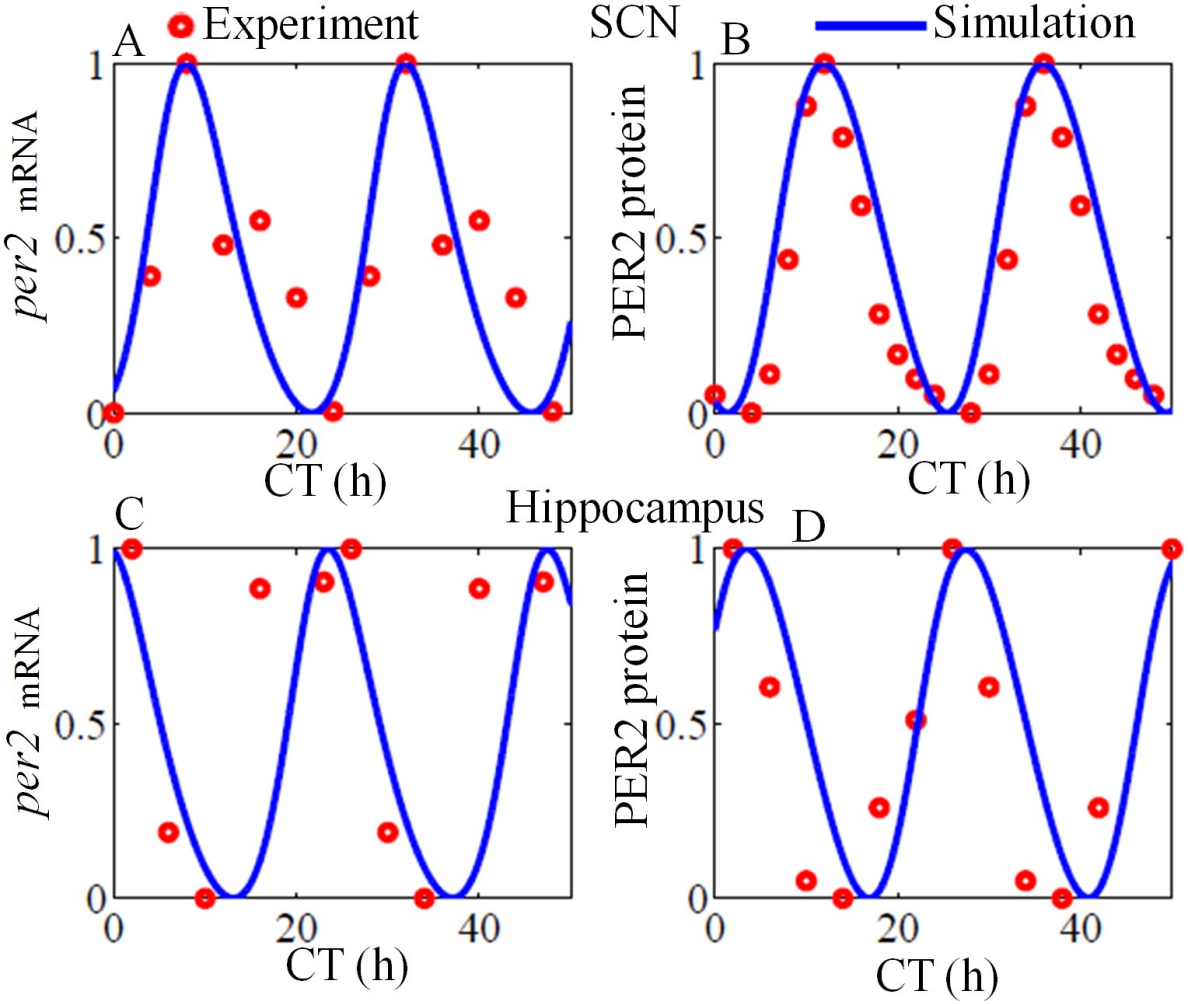
Free running oscillation at SCN and hippocampus under constant darkness condition. Blue curves are from simulation, and red circles are the experimental data points. Simulated *per2* mRNA and protein at SCN (A, C) and hippocampus shows antiphase oscillations, which is good agreement with the experimental observations. Simulation results were obtained by integrating the model equations given in materials and method section with *I*_*pre*_ = 0. For comparison, the individual time series were normalized to [0, 1]. Experimental data points of *per2* mRNA and protein at SCN are extracted from [60] and for hippocampus, the data is extracted from [10].

**Fig 6:**
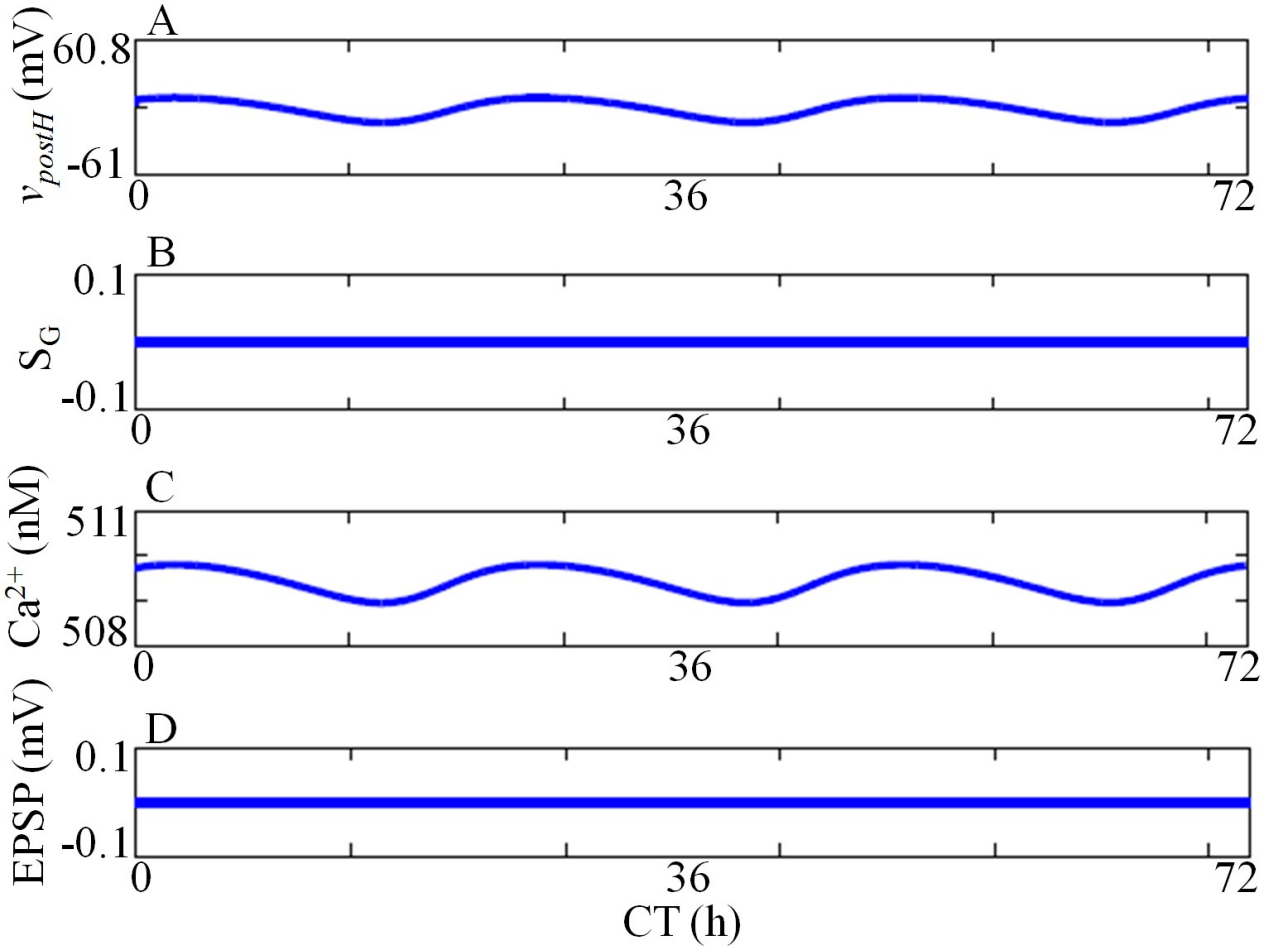
Circadian variation of SCN-hippocampus model variables without any pre-post activity. *v*_*postH*_ and calcium concentrations shows circadian variation (A, C), and the corresponding time series of *S*_*G*_ (B) and EPSP (D).

Experimental observations suggest that hippocampal LTP/LTD, learning and memory are regulated by circadian rhythm [14, 19]. Rawashdesh et al. [12] suggest that *per1* controls hippocampal rhythm and memory. Wang et al. [10] reported the rhythmic expression of *per2* mRNA and protein at hippocampus. Therefore we speculate that there is a direct link between SCN and hippocampus that regulate LTP/LTD, learning and memory. In our model, we introduce Goodwin type model for SCN that diffusively couples to hippocampus GRN via an intermediate neurotransmitter that is yet unknown. The free running oscillations of SCN and hippocampus GRN is shown in Fig 5. We fit the model to the experimentally observed *per2* mRNA and protein data [10, 60]. Under DD condition (*L* = 0), both the oscillators have a free running period of 23.6h, but the hippocampal oscillation is antiphase with SCN. This is in good agreement with the experimental observation [10]. In our model, we assume that NMDAR conductance is controlled by the hippocampal GRN variable *P*_1*Ph*_. The resulting oscillations of other variables in the absence of pre-postsynaptic activities are shown in Fig 6.

**Fig 7:**
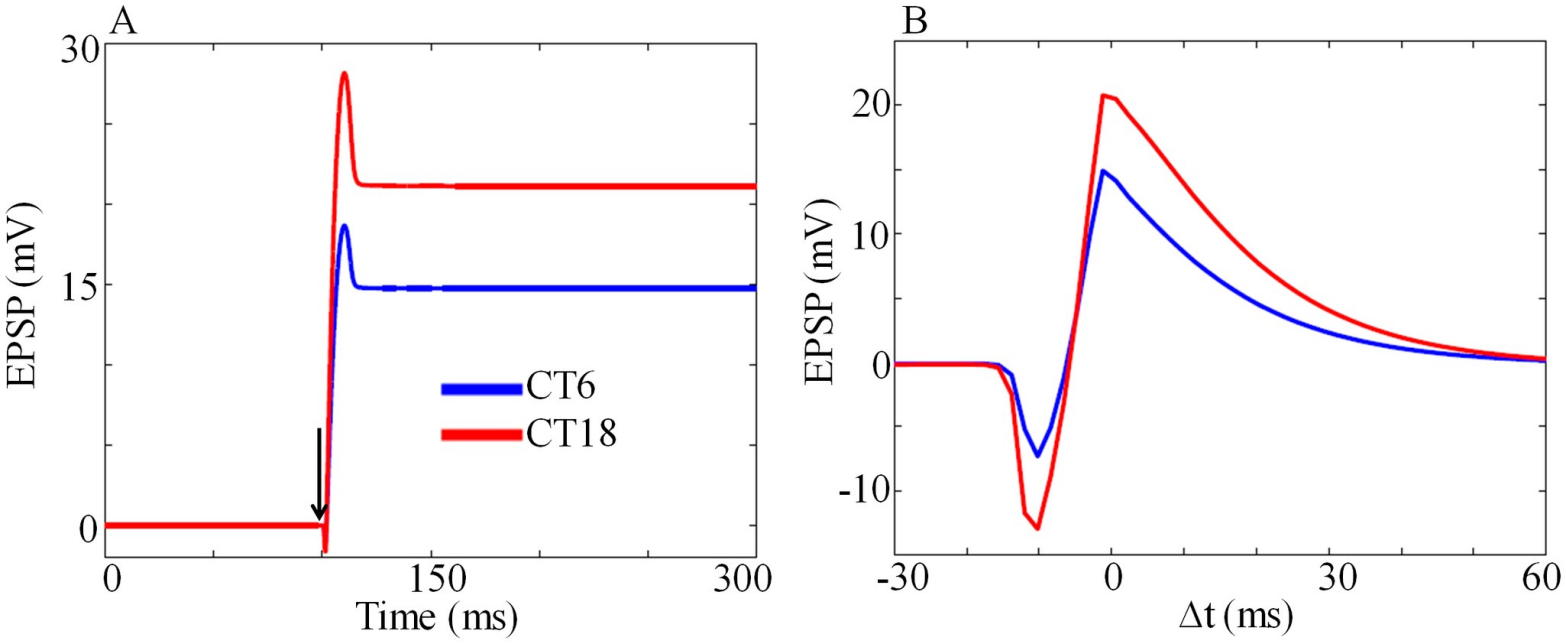
LTP at two different circadian times. (A) *EPSP* as a function of time at subjective day (CT6) and subjective night (CT18), where Δ*t* = 0. Larger LTP in simulation seen during subjective night is in good agreement with the experimental results [19]. Arrow head indicate the time at which *I*_*post*_ and *I*_*pre*_ are applied. (B) EPSP as a function of Δ*t*. The magnitude of the *I*_*pre*_ and *I*_*post*_ are 35 pA and 800 pA respectively, and the duration of the current is 2*ms*.

Now we simulate the circadian dependents of LTP and LTD by introducing pre and postsynaptic spikes. We calculate the EPSP change at two different circadian phases, one at subjective day (CT6) and the other at subjective night (CT18). LTP at subjective night is higher than that of during the subjective day (Fig 7A) as seen in the experiments [19]. Again we simulate the complete STDP curve that also shows circadian variation (Fig 7B). Maximum LTP that occurs at subjective night is larger in comparison to LTP during subjective day.

#### 2.2.3 Circadian variation of LTP/LTD under LD condition

**Fig 8:**
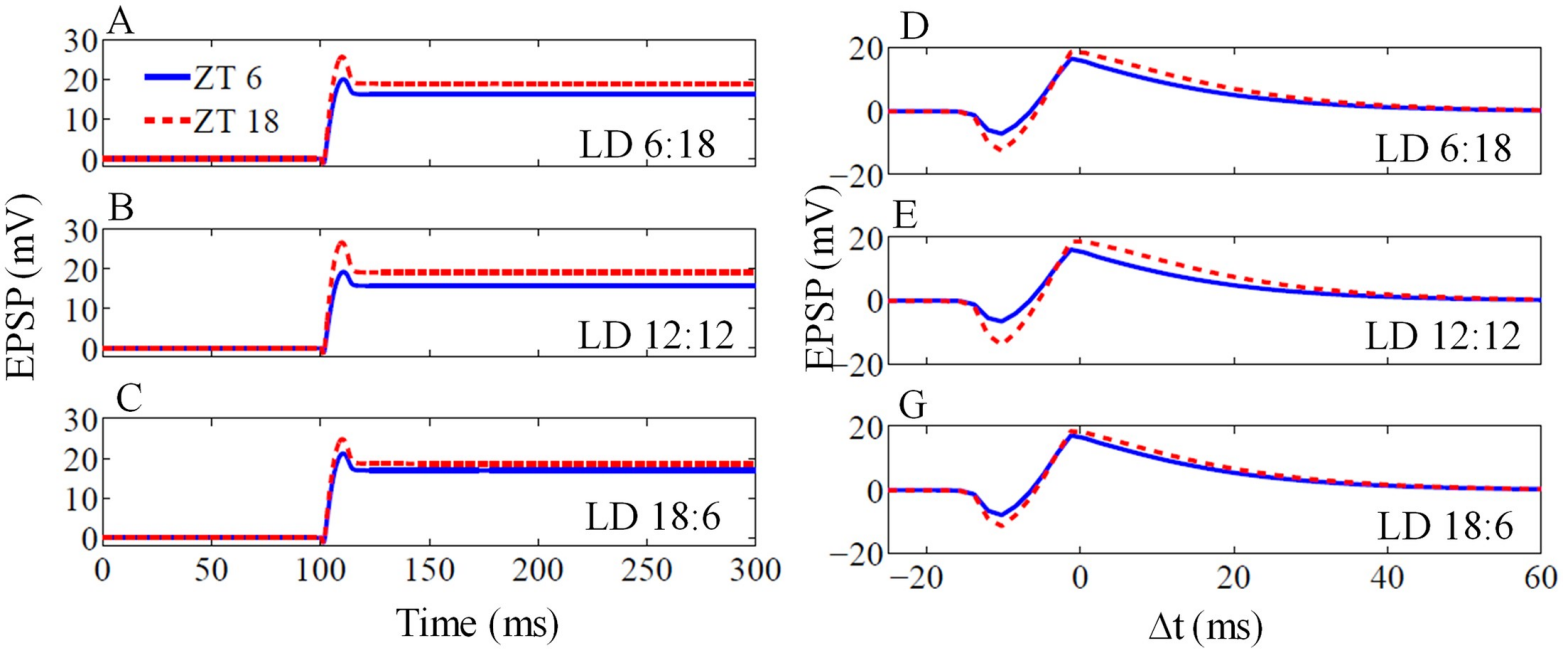
LTP and STDP under different LD cycles. (A-C) LTP for ZT6 (blue line) and ZT18 (red broken line) under different LD cycle with Δ*t* = 0. For all LD case, LTP during subjective night (ZT18) is higher than subjective day (ZT6). (D-E) STDP curves obtained at ZT6 and ZT18 under different photoperiods. For all the LD case, the magnitude of EPSP amplitude during subjective night is higher than the subjective day.

We test the synaptic plasticity under LD conditions with different photoperiods. We compute STDP under different photoperiods ((Fig 8), and in all the photoperiods that we consider, the maximum of LTP amplitude occur during night (ZT18), which is larger in comparison to the day (ZT6). This is in good agreement with that experimental results [19], which shows the LTP magnitude is higher at night than during day.

### 2.3 Circadian modulation of learning and memory at amygdala

#### 2.3.1 Circadian rhythm modulates acquisition

We test whether our model can learn to simulate the fear conditioning protocol. We first explain the parameters that we use in the model to simulate the CS-US pairings and a unique parameter that we use to capture the percent freezing response in the experiments. Here, we take the spike at the postsynaptic neuron (*v*_*postA*_) responsible for fear response. We consider postsynaptic stimulus (*I*_*post*_) as US, which induces spike in postsynaptic neuron. We take CS as the combination of pre-synaptic stimulus (*I*_*pre*_) and *I*_*post*_ to train the post-synaptic neuron to generate a spike in an associative fashion. Whenever *I*_*pre*_ is applied, pre-synaptic neuron release glutamate that binds to both NMDAR and AMPAR. *I*_*post*_ increase the membrane potential in postsynaptic neuron and produces a spike. If we apply *I*_*pre*_ and *I*_*post*_ simultaneously, NMDAR conduction induces more amount of *Ca*^2+^, and this NMDAR evoked calcium current in turn activate more amount of AMPAR, which eventually increase the AMPAR conductance (*g*_*AMPAR*_) to increase the overall synaptic strength. Thus, the paired exposure of CS and US cause LTP, the electrophysiological measure of learning and memory. In the model, we take AMPAR conductance, *g*_*AMPAR*_, to compare with the freezing percentage of experimental fear response data.

**Fig 9:**
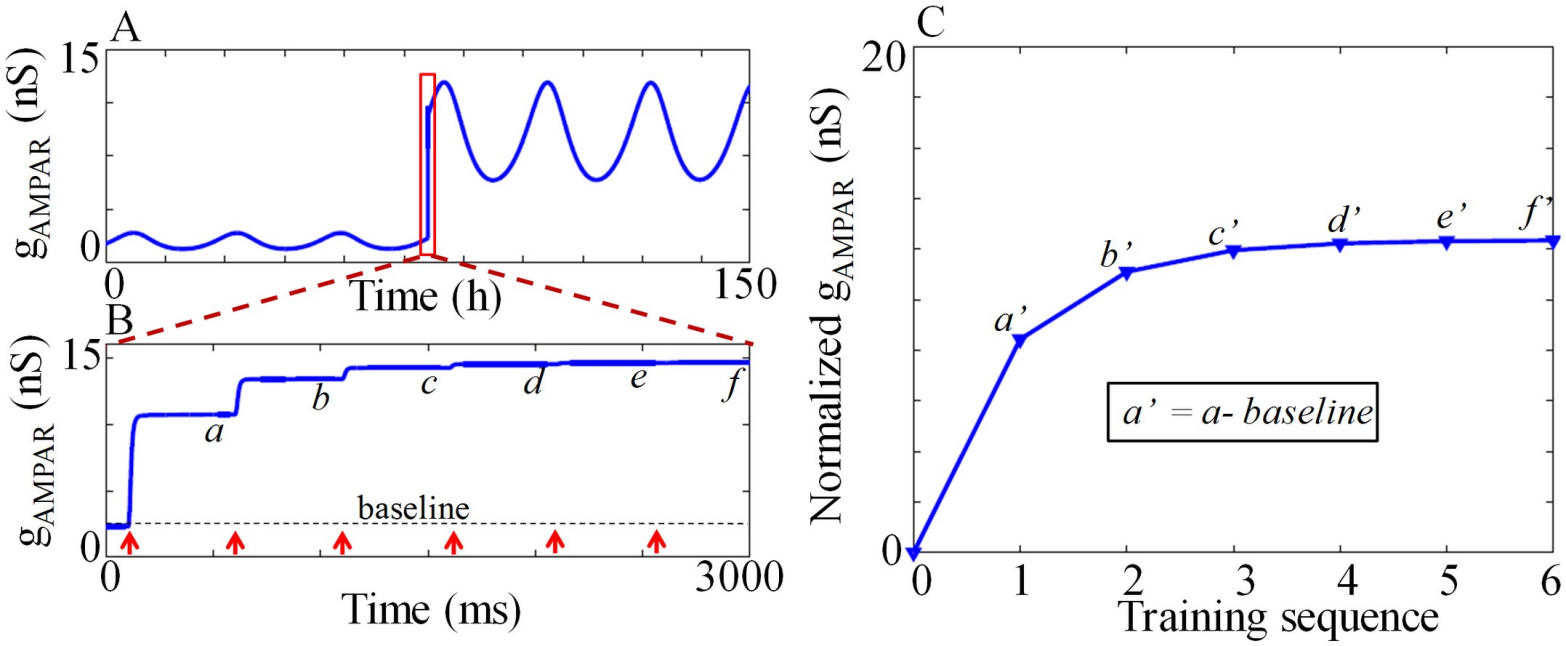
Dynamics of *g*_*AMPAR*_. (A)*g*_*AMPAR*_ as a function of time, which varies in a circadian manner. Model is trained by applying 6 pairs of *I*_*pre*_ (CS) and *I*_*post*_ (US) at CT3 (CT 75 *mod* 24), which increases the *g*_*AMPAR*_ level. (B) Enlarged view of the application of 6 pairs of *I*_*pre*_ and *I*_*post*_ with an inter-trial interval of 500*ms*. Red arrows indicate the time at which each *I*_*pre*_ -*I*_*post*_ pair we appy. To determine the degree of learning, we use *g*_*AMPAR*_ level at the end of the each inter-trial interval (*a, b, c, d, e, f*). (C) Normalized *g*_*AMPAR*_ value for each training sequence. *a’, b’, c’, d’, e’, f’* are the corresponding normalized values of *a, b, c, d, e, f* in (B). Normalized *g*_*AMPR*_ is obtained by subtracting the baseline value from the original value.

In experiments conducted with C-3H mice to find the circadian modulation of acquisition, Chaudhury et al. [14] applied 6 pairs of CS-US with an inter-trial interval of 64s. To determine the degree of learning, they calculated the percentage of freezing. In that experiment, US was taken as 1mA footshock and CS was taken as the tone or context. To mimic the experimental procedure in [14] in our simulation, our model training protocol consists of six *I*_*pre*_-*I*_*post*_ pairing with 35 *pA* *I*_*pre*_ and 400 *pA* *I*_*post*_ with an inter-trial interval of 500*ms*. We take the *g*_*AMPAR*_ level to calculate the degree of learning (acquisition) as shown in Fig 9. Circadian variation of *g*_*AMPAR*_ under 12:12 LD cycle is shown Fig 9A. Application of *I*_*pre*_-*I*_*post*_ increases AMPAR, and hence the *g*_*AMPAR*_. Enlarged view of *g*_*AMPAR*_ during training is shown in Fig 9B. To determine the degree of learning, we calculate the normalized *g*_*AMPAR*_ value for each training sequence (Fig 9C) by subtracting the baseline value from the original value we obtain after the training. Normalized *g*_*AMPAR*_ value *a’, b’, c’, d’, e’, f’* in Fig 9C are the corresponding normalized values of *a, b, c, d, e, f* in Fig 9B.

**Fig 10:**
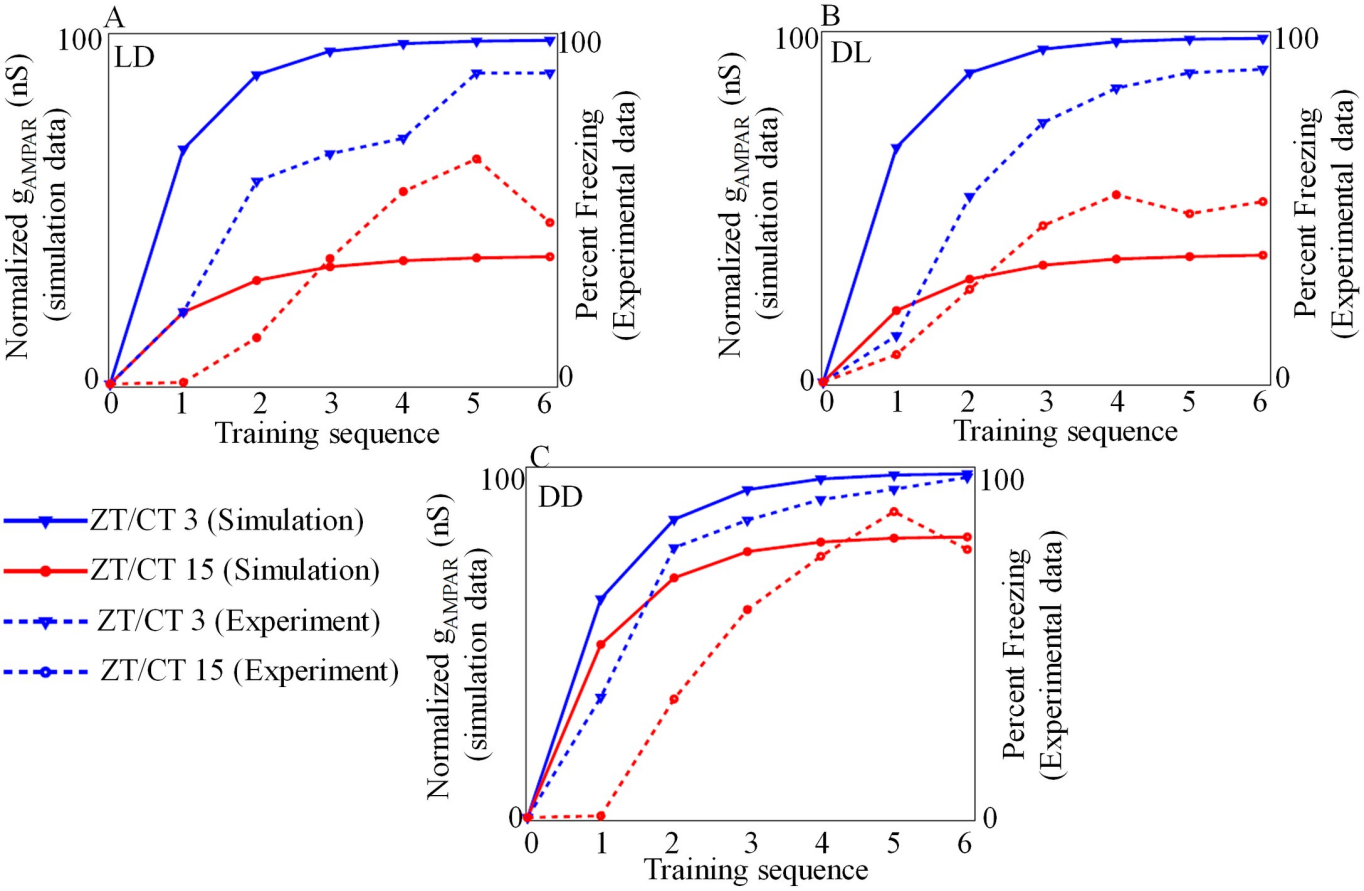
Acquisition. Model is trained by applying *I*_*pre*_ (CS) and *I*_*post*_ (US) either in the day (ZT/CT 3) or in the night (ZT/CT 15). The model trained in the daytime acquire better synaptic strengthening (*g*_*AMPAR*_ increment) than trained at night. (A) Model forced by 12:12 LD cycle (B) model forced by 12:12 DL cycle (C) model is under DD condition. In all cases *I*_*pre*_, *I*_*post*_ are 35mA, 400mA, respectively with a duration of 2*ms*. Normalized *g*_*AMPR*_ is obtained by subtracting the baseline value from the original value and minimum value of *g*_*AMPR*_ is normalized to 0 and maximum value is normalized to 100. This enabled better comparison of experimental and simulation data points, and it is clear that degree of acquisition is more during day. Baseline value for ZT/CT3 is 2.3 and for ZT/CT15 is 1.3. Experimental data points are extracted from [14].

To test the circadian rhythm during acquisition, we conduct 3 experiments as shown in Fig 10. In the first experiment, we subject the model to 12:12 LD cycle and then we apply CS-US pair at ZT3 (day) and another at ZT15 (night). ZT0 is the taken as light onset. The degree of acquisition captured by *g*_*AMPAR*_ shows high increment in conductance when we train during subjective day (Fig 10A). In the second experiment, we force the model to 12:12 DL cycle (reversed LD), and we find that the degree of acquisition is still higher during the subjective day (Fig 10B) as seen in the 12:12 LD cycle. To test whether the acquisition is modulated by circadian rhythm or controlled by only LD cycle, we again train the model under DD condition, and the results are shown in Fig 10C. In DD condition, from simulation, we find the acquisition is higher during the subjective day (CT3) than during the subjective night (CT15). Calculated day and night differences in the degree of acquisition which are provided in the S4 Table, shows that there is a significant difference in acquisition between ZT/CT 3 and ZT/CT 15. Even though our model fails to capture the exact trend in the degree of acquisition as seen in the experiments [14], qualitatively the trend is captured it is clear that acquisition is modulated by circadian rhythm.

### 2.4 Circadian rhythm modulates recall

**Fig 11:**
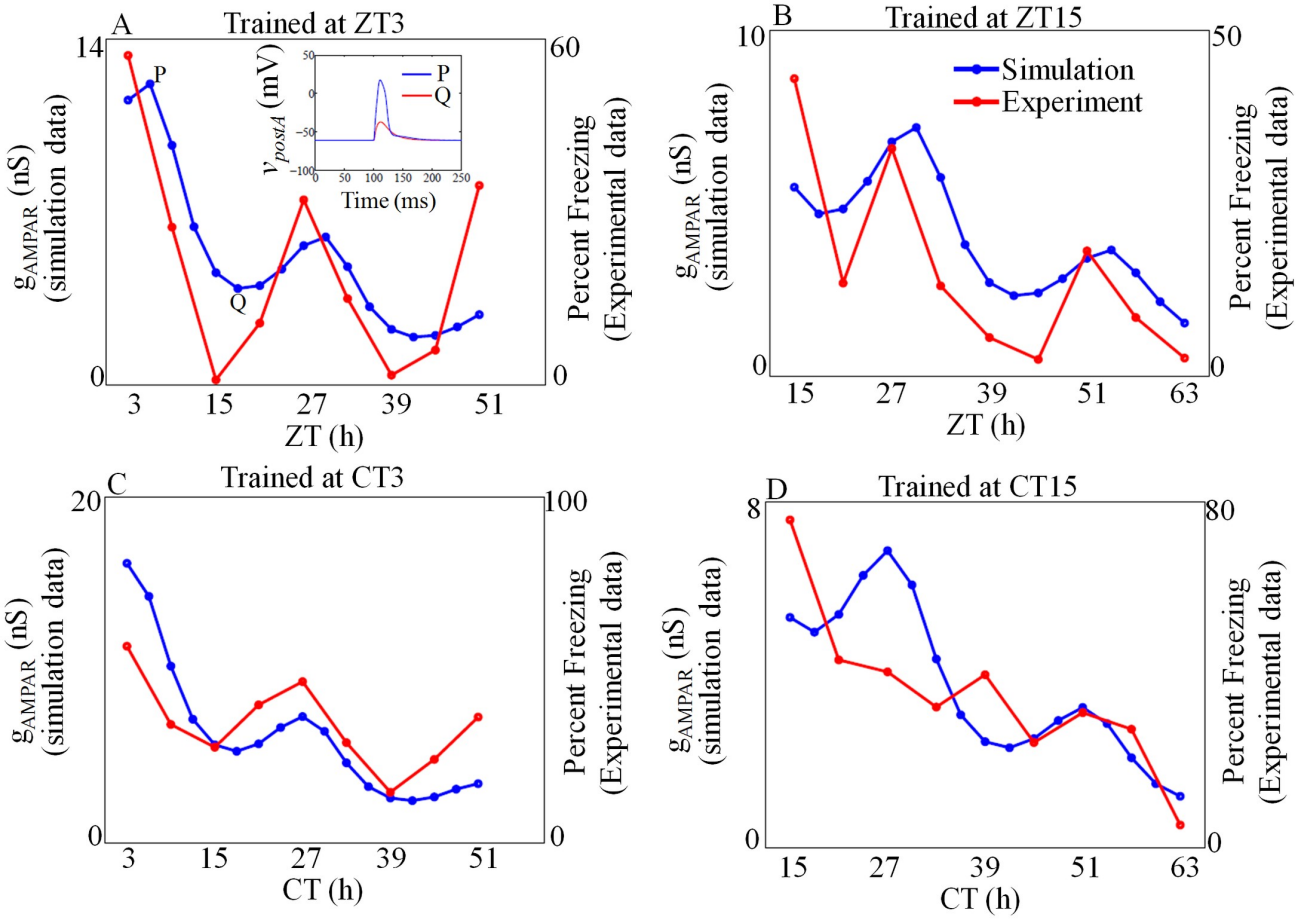
Recall. The model trained either in the day (ZT/CT 3) or night (ZT/CT 15) is tested for recall. The model shows better recall during the day, irrespective of the time of training. (A, B) Model forced by 12:12 LD cycle (C, D) model is under DD condition. Sample spikes generated during the recall test is shown in the inset of A. For training six pairs of *I*_*pre*_ and *I*_*post*_ were applied and for recall, a single *I*_*pre*_ pulse was applied. Magnitude of *I*_*pre*_ and *I*_*post*_ are 35mA, 400mA respectively with a duration of 2*ms*. Experimental data points are extracted from [14].

After conditioning, we test whether the model can simulate recall at different circadian phases. Before conditioning, postsynaptic neuron initially responds to the *I*_*post*_ (US) by producing an action potential. After conditioning the neuron, it eventually respond to *I*_*pre*_ (CS). However, the efficiency of response (recall) is varied as a function of the time-of-the-day in which the recall test is performed. In the recall test, we apply only *I*_*pre*_ to generate a spike in the post-synapse, and the corresponding *g*_*AMPAR*_ values are shown in Fig 11. In the first test, when we train the model at ZT3 under 12:12 LD cycle, we test for recall every 3h starting from 24h after the training (Fig 11). The *g*_*AMPAR*_ value peaks during the daytime in each cycle (ZT6, ZT30) and reaches minimum value during the night time (ZT18, ZT42). Two sample spikes generated during recall test is shown in Fig 11A inset. A proper spike is generated at ZT6, but only a subthreshold spike is generated at ZT18, and this indicates that our model obtain better recall during the daytime. We also simulate the recall test trained during the night under 12:12 LD cycle and the results are shown in Fig 11B. Again, the maximum values of *g*_*AMPAR*_ are seen during the daytime, and we only obtain minimum values during the night time. In order to test whether the recall is modulated by circadian rhythm or controlled by only LD cycle, we train and test the model only under DD conditions, and the results are shown in Fig 11C and 11D. When we conduct the recall test at different circadian time, one at subjective day (CT3) and the other at subjective night (CT15), recall process still persists with greater degree during the daytime (Fig 11C and 11D).

### 2.5 Circadian rhythm modulates extinction

**Fig 12:**
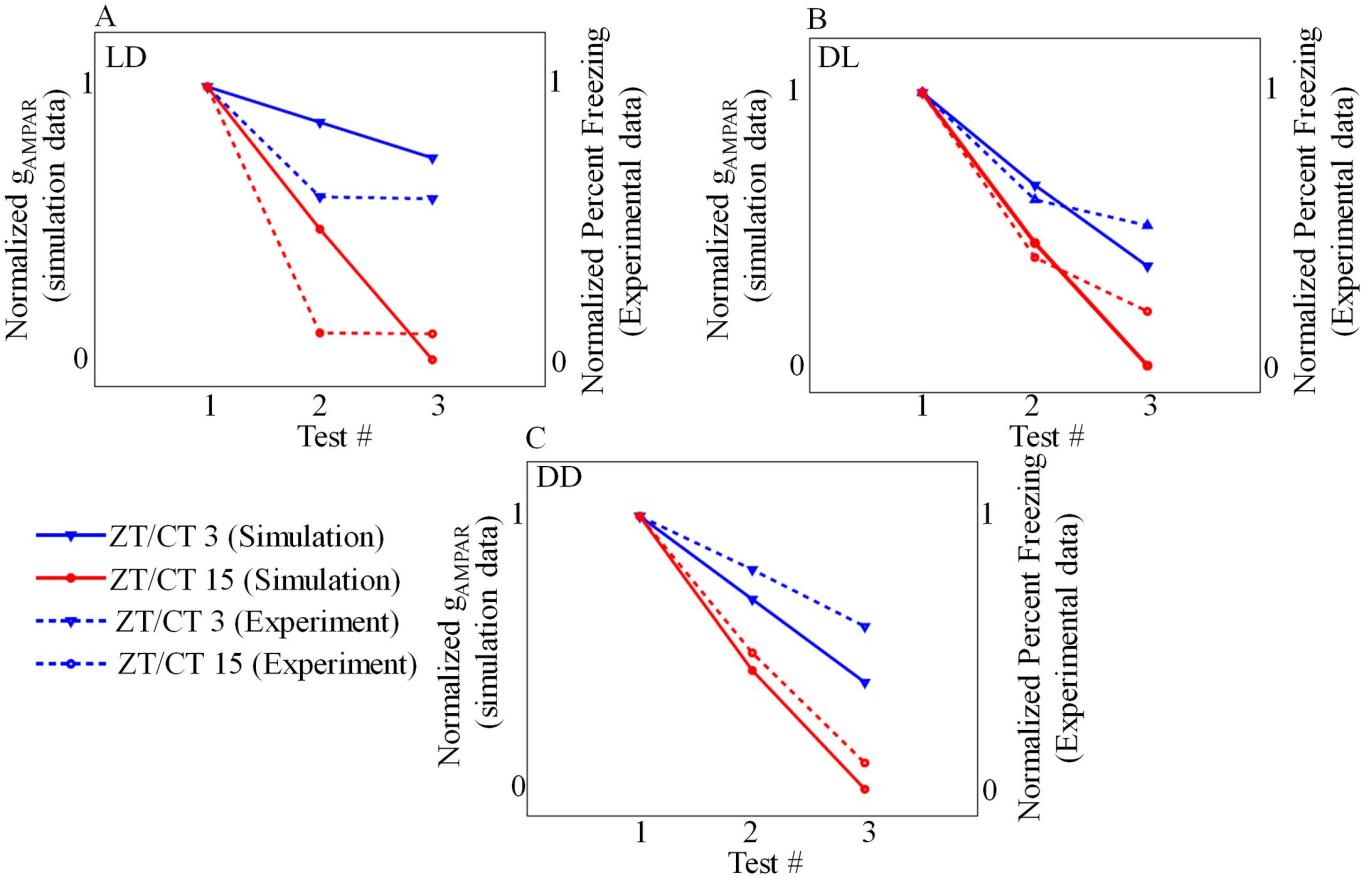
Extinction. Model is trained and tested for extinction either in day or night. Degree of extinction is higher in model trained and tested at night (A-C, red triangle). (A) Model forced by 12:12 LD cycle (B) model forced by 12:12 DL cycle (C) model is under DD condition. For comparison, *g*_*AMPAR*_ values obtained after the first extinction test at day and night are normalized to 1 and minimum value is normalized to 0. it is clear that degree of extinction is more during night. Experimental data points are extracted from [14].

Repeated application of CS will decrease the synaptic strength, and as a result, it decreases the conditioned response. We test in our simulation the extinction property by applying repeated *I*_*pre*_ at different time of the day, and the results are shown in Fig 12. In the first test, we train and test the model either during the day (ZT3) or night (ZT15) under LD conditions. We found that the model trained and tested during night shows a greater degree of extinction (Fig 12A). We obtain similar results even in the reversed LD cycle (DL) (Fig 12B). Finally, when we train and test the model during subjective night, the model exhibit greater degree of extinction under DD condition (Fig 12C). Day and night difference in degree of extinction is calculated and it is given in S4 Table, which shows that there is a significant difference in extinction between ZT/CT 3 and ZT/CT 15. These results once again reinforce that extinction modulated by circadian rhythm is higher during the night, and this is in confirmation with the experiments [14]. Even though our model fails to reproduce the exact trend in the degree of extinction as seen in the experiments [14], the trends in the simulation is similar to the one seen in the experiments. These results suggest that extinction is modulated by circadian rhythm.

## 3 Discussion and conclusion

In this work, through multi-scale modeling, we showed that the circadian rhythm modulates learning and memory. Previous studies have shown that SFR at SCN exhibited circadian oscillation with a period close to 24h. In this study, we incorporated both GRN and conductance model of SCN to show the circadian modulation of SFR. Importantly, we showed the circadian modulation of voltage-gated calcium current (VGCC) played an important role in the modulation of SFR that peaks during day and reach nadir during night.

Though the impact of circadian rhythm on LTP/LTD and memory has long been studied from the molecular mechanism point of view, it is not well understood how circadian rhythms modulated LTP/LTD, and memory formation. However, previous studies [10–12] provided information about how *per* genes (*per1, per2*) influenced the synaptic modification and acted as a molecular link between circadian rhythms and synaptic plasticity. In this work we related *per2* in the hippocampus with synaptic plasticity and obtained experimentally observed circadian phase-dependent variation of LTP/LTD under both DD and LD conditions. In our model, calcium that entered the postsynaptic neuron through NMDAR is the key signal for the simulation of synaptic plasticity. Another important regulation we made in our model is that the conductance of the NMDAR channel is controlled by the hippocampal *per2*. Thus the *Ca*^2+^ dynamics due to the pre and postsynaptic activity is made circadian phase-dependent with the result that it played a strong role in synaptic plasticity.

Based on earlier works, we considered SCN and hippocampus as a master-slave oscillator. However, hippocampal circadian genes showed anti-phase oscillation with respect to SCN GRN’s. Presently, how this anti-phase oscillation influences the circadian modulation of LTP/LTD is not known. Simulation results showed variation in LTP and STDP at different circadian phases (Fig 7) under DD condition. Maximum EPSP change happened at subjective night, is in good agreement with the experimental observations [19]. We also tested the model under LD condition, where it showed maximal EPSP change occurred during night time and this simulated results agreed well with the experimental findings [54]. Eckel-Mahan et al. [84] suggested that the memory training may work as zeitgeber for hippocampal neurons. They reported that memory training-induced signaling at hippocampus and light-induced molecular events at SCN are comparable. The phase response curve (PRC) of the mammalian system explained the circadian phase-dependent transient changes in the system in response to perturbation (generally light). The phase responses during the subjective day are different from the subjective night. Similarly, LTP induced by pre-postsynaptic activity also changed in a circadian phase-dependent manner, and our mathematical model faithfully captured this aspect.

Finally, we presented a multi-scale model of the SCN-amygdala to investigate the mechanism behind the circadian modulation of learning and memory at amygdala. We simulated fear conditioning, recall, and extinction at a different time of day and our model qualitatively reproduced the experimental results [14]. The acquisition is maximum when the model is trained during the day, the recall ability is maximum during the day irrespective of the time of training, and the degree of extinction is higher when the model is trained and tested during the night. All these are in good agreement with the experimental results [14]. Experimental studies suggest that activation of NMDAR in the amygdala is necessary for fear conditioning [85], and AMPAR trafficking occurs to synapse during learning [77], and our model used in the simulation also consists of coupled GRN network of SCN and amygdala along with the electrophysiology of spiking neuron and the dynamics of AMPAR, NMDAR, and calcium. In this work, the addition and removal of AMPAR, the conductance of NMDAR, AMPAR, and calcium channel are modeled in such a way that their dynamics oscillate with a circadian period. This modeling approach allowed us to explore the circadian modulation of fear conditioning, recall, and extinction at amygdala. Our model study strongly supports the NMDAR evoked calcium current and AMPAR plays an important role in fear conditioning, and the circadian variation of their dynamics eventually modulated learning and memory.

In summary, our model captures the essence of circadian modulation of learning and memory. However, there are many aspects of this modelling task that we undertook require refinement and modifications. Firstly, since we didn’t consider the population of neurons for modelling, the dynamics of population of neurons in SCN, hippocampus, and amygdala may suggest otherwise. Secondly, electrophysiological models that we used in this study are simple, and hence most of the simulation results can only be qualitatively matched with the experimentally observed trends. Thirdly, we focused only on the mice models and have not considered the learning and memory aspects that are studied extensively in other models like *Drosophila*. Finally, our GRN model, though considered *per2*/PER2 dynamics, is very minimalistic. Since our aim is to understand the relationship between circadian dynamics and learning, as a first go, we confined ourselves to a very simple phenomenological model. Also, there is a lack of molecular mechanisms driving these processes and most of the experimental data relating circadian influence on fear conditioning paradigm cannot be correlated to molecular mechanisms. In this work, we assumed most of the molecular mechanisms and therefore, resorted to simple molecular models. We could have considered more realistic models like morning and evening (ME) oscillators [30], where it explained how circadian clock genes control morning and evening activities, but again to know how the GRN model communicates with the conductance model through neurotransmitters or other coupling agents is a big challenge. This can be at best speculated to model the process. All these limitations can be addressed in the future work only after getting sufficient clarity from the experiments which can provide sufficient insight into the molecular mechanisms operating between the GRN’s, channel dynamics and neurotransmitters.

## Supporting information

S1 Table Model for circadian modulation of SFR at SCN. (PDF)

S2 Table Model for circadian modulation of LTP/LTD at hippocampus. (PDF)

S3 Table Model for circadian modulation of learning and memory at Aygdala. (PDF)

S4 Table Day and night difference in degree of acquisition, and extinction. (PDF)

S1 Data Data for *per*2 gene and protein at SCN and hippocampus. (XLSX)

S2 Data Data for acquisition, recall and extinction. (XLSX)

S1 Program Xppaut files for programs. (ZIP)
