# Supplementary material for: Multi-scale modeling of the circadian modulation of learning and memory
Source: PLoS One. 2019 Jul 19;14(7):e0219915. doi: 10.1371/journal.pone.0219915 (PMC6641212; doi:10.1371/journal.pone.0219915)
Supplement: S1 Table — (PDF) [file pone.0219915.s001.pdf]

S1 Table. Model for circadian modulation SFR at SCN

Model	Parameters
**GRN for SCN**	
$\frac{d}{dt}M_{Ps} = A_s(v_{s1}\frac{K_{As}^{n_c}}{K_{As}^{n_c}+P_{1s}^{n_c}} - M_{Ps}) + L$<br>$\frac{d}{dt}P_{1s} = A_s(M_{Ps} - P_{1s})$<br>$\frac{d}{dt}P_{1Ps} = A_s(P_{1s} - P_{1Ps})$	$A_s = 4.35e-8\ ms^{-1}$, $v_{s1} = 20\ nM$, $K_{As} = 0.8\ nM$,<br>$n_c = 9, L = 0$
**Modified ML model**	
$C\frac{d}{dt}v = I_{app} - I_L - I_k - I_{Ca}$<br>$= I_{app} - g_L(v - v_L) - g_K w(v - v_k) - g_{Ca}m_\infty(v - v_{Ca})$<br>$\frac{dw}{dt} = \lambda(w_\infty - w)$<br>$m_\infty = 0.5(1 + tanh(\frac{v-v_1}{v_2}))$<br>$w_\infty = 0.5(1 + tanh(\frac{v-v_3}{v_4}))$<br>$\lambda = \phi cosh(\frac{v-v_3}{2v_4})$<br>$g_{Ca} = g_{cabase}\frac{M_{ps}}{k_{ps}+M_{ps}}$	$C = 20\ pF$, $g_L = 2\ nS$, $g_K = 30\ nS$, $v_k = -84mV$, $v_{Ca} = 90mV$,<br>$v_L = -60mV$, $v_1 = -1.2mV$, $v_2 = 18mV$,<br>$v_3 = 12mV$, $v_4 = 14.75mV$, $\phi = 0.04$<br>$g_{cabase} = 6.37\ nS$, $k_{ps} = 0.1\ nM$
