# Supplementary material for: Multi-scale modeling of the circadian modulation of learning and memory
Source: PLoS One. 2019 Jul 19;14(7):e0219915. doi: 10.1371/journal.pone.0219915 (PMC6641212; doi:10.1371/journal.pone.0219915)
Supplement: S2 Table — (PDF) [file pone.0219915.s002.pdf]

S2 Table. Model for circadian modulation of LTP/LTD at HC

Model	Parameters
**GRN for HC**	
$\frac{d}{dt}M_{Ph} = A_s(v_{s2}\frac{K_{Ah}^{nc}}{K_{Ah}^{nc}+P_{1h}^{nc}} - M_{Ph}) + v_{ss}\frac{R_{C1}^{nc}}{K_c^{nc}+R_{C1}^{nc}} + K_{scr}S_{CREB}CREB$<br>$\frac{d}{dt}P_{1h} = A_s(M_{Ph} - P_{1h})$<br>$\frac{d}{dt}P_{1Ph} = A_s(P_{1h} - P_{1Ph})$	$A_s = 4.35e-8\ ms^{-1}$<br>$v_{s2} = 20\ nM$, $K_{Ah} = 0.8\ nM$, $v_{ss} = 1e-3\ nMms^{-1}$<br>$k_{scr} = 1e-4\ ms^{-1}$, $K_c = 5\ nM$, $n_c = 9$
**Coupling between SCN, HC, and Ca^{2+}**	
$\frac{d}{dt}R_{C1} = (P_{1ps} - R_{C1})$<br>$\frac{d}{dt}S_{CREB} = a_rI_{pre}(1 - S_{CREB}) - a_dS_{CREB}$	$a_r = 0.01\ pA^{-1}ms^{-1}$, $a_d = 0.07\ ms^{-1}$
**NMDA Current**	
$I_{NMDA} = g_{NMDA}S_GB_v(v_{postH} - v_{NMDA})$<br>$g_{NMDA} = g_N\frac{k_{norm}^{nm}}{P_{1ph}^{nm}}$<br>$B_v = \frac{1}{1+0.005[Mg^+]e^{-0.2v_{postH}}}$<br>$\frac{d}{dt}S_G = a_rI_{pre}(1 - S_G) - a_dS_G$	$v_{NMDA} = 0\ mV$, $g_N = 12\ nS$, $k_{norm} = 1\ nM$, $n_m = 2$<br>$Mg^+ = 1$
**Modified ML model**	
$C\frac{d}{dt}v_{postH} = I_{post} - I_L - I_k - I_{Ca} - I_{NMDA}$<br>$= I_{post} - g_L(v_{postH} - v_L) - g_kw(v_{postH} - v_k) - I_{NMDA}$<br>$\frac{dw}{dt} = \lambda(w_\infty - w)$<br>$m_\infty = 0.5(1 + tanh(\frac{v_{postH}-v_1}{v_2}))$<br>$w_\infty = 0.5(1 + tanh(\frac{v_{postH}-v_3}{v_4}))$<br>$\lambda = \phi cosh(\frac{v_{postH}-v_3}{2v_4})$	$C = 20\ pF$, $g_L = 2\ nS$, $g_K = 8\ nS$, $v_k = -84mV$, $v_{Ca} = 120mV$,<br>$v_L = -60mV$, $v_1 = -1.2mV$, $v_2 = 18mV$,<br>$v_3 = 2mV$, $v_4 = 30mV$, $\phi = 0.08$
**Calcium dynamics**	
$g_{Ca} = g_{cabase}\frac{M_{ph}}{k_{ph}+M_{ph}}$<br>$\frac{d}{dt}Ca = k_{NMDA}I_{NMDA} - k_{Ca}I_{Ca} + \frac{Ca_0-Ca}{\tau_{Ca}}$	$g_{cabase} = 4.35\ nS$, $k_{ph} = 0.1\ nM$<br>$Ca_0 = 500nM$, $k_{INMDA} = 1$, $k_{Ca} = 1$, $\tau_{Ca} = 10ms$
**EPSP and AMPAR dynamics**	
$\frac{d}{dt}EPSP = I_{NMDA}/C$<br>$\frac{d}{dt}AMPAR = (a_{r1}(Ca - Ca_1)(A_m - AMPAR) - a_{d1}AMPAR(Ca - Ca_1))S_{AMPA}$<br>$\frac{d}{dt}S_{AMPA} = a_{rs}I_{post}(1 - S_{AMPA}) - a_{ds}S_{AMPA}$	$a_{r1} = 1e-7\ nM^{-1}ms^{-1}$, $A_m = 1e4$, $a_{d1} = 1e-5\ nM^{-1}ms^{-1}$,<br>$a_{rs} = 0.01\ pA^{-1}ms^{-1}$, $a_{ds} = 0.0007\ ms^{-1}$, $Ca_1 = 509.5\ nM$
