# Supplementary material for: Multi-scale modeling of the circadian modulation of learning and memory
Source: PLoS One. 2019 Jul 19;14(7):e0219915. doi: 10.1371/journal.pone.0219915 (PMC6641212; doi:10.1371/journal.pone.0219915)
Supplement: S3 Table — (PDF) [file pone.0219915.s003.pdf]

S3 Table. Model for circadian modulation of learning and memory at Aygdala

**Model**	**Parameters**
**GRN for Amygdala**	
$\frac{d}{dt}M_{pa} = A_s(v_{s2}\frac{K_{Ah}^{nc}}{K_{Ah}^{nc}+P_{1a}^{nc}} - M_{pa}) + v_{ss}\frac{K_I^{nc}}{K_c^{nc}+R_{C2}^{nc}}$<br>$\frac{d}{dt}P_{1a} = A_s(M_{pa} - P_{1a})$<br>$\frac{d}{dt}P_{1pa} = A_s(P_{1a} - P_{1pa})$	$A_s = 4.35e-8\ ms^{-1}$, $n_c = 2$<br>$v_{s2} = 20\ nM$, $K_{Ah} = 0.8\ nM$, $v_{ss} = 1e-3\ nMms^{-1}$<br>$k_{scr} = 1e-4$, $K_c = 5\ nM$,
**Coupling between SCN and Amygdala**	
$\frac{d}{dt}R_{C2} = (P_{1ps} - R_{C2})$	
**NMDAR, AMPAR Current**	
$I_{NMDA} = g_{NMDA}S_G B_v(v_{postA} - v_{NMDA})$<br>$g_{NMDA} = g_N\frac{k_{norm}^{nm}}{P_{1pa}^{nm}}$<br>$B_v = \frac{1}{1+0.005[Mg^+]e^{-0.2v_{postA}}}$<br>$\frac{d}{dt}S_G = a_r I_{pre}(1-S_G) - a_d S_G$<br>$I_{AMPAR} = g_{AMPAR}S_G(v_{postA} - v_{AMPA})$<br>$g_{AMPAR} = g_{AM}\frac{k_{am1}^{rr}}{P_{1pa}^{rr}}AMPAR$	$v_{NMDA} = 0\ mV$, $g_N = 12\ nS$, $k_{norm} = 1\ nM$, $n_m = 2$<br>$Mg^+ = 1$<br>$a_r = 0.01\ pA^{-1}ms^{-1}$, $a_d = 0.07\ ms^{-1}$<br>$v_{AMPA} = 0\ mV$<br>$g_{AM} = 0.2\ nS$, $k_{am1} = 1.3\ nM$, $r_r = 3$
**Modified ML model**	
$C\frac{d}{dt}v_{postA} = I_{post} - I_L - I_k - I_{Ca} - I_{NMDA} - I_{AMPAR}$<br>$= I_{post} - g_L(v_{postA} - v_L) - g_k w(v_{postA} - v_k) -$<br>$g_{Ca}m_\infty(v_{postA} - v_{Ca}) - I_{NMDA} - I_{AMPAR}$<br>$\frac{dw}{dt} = \lambda(w_\infty - w)$<br>$m_\infty = 0.5(1+tanh(\frac{v_{postA}-v_1}{v_2}))$<br>$w_\infty = 0.5(1+tanh(\frac{v_{postA}-v_3}{v_4}))$<br>$\lambda = \phi cosh(\frac{v_{postA}-v_3}{2v_4})$	$C = 20\ pF$, $g_L = 2\ nS$, $g_K = 8\ nS$, $v_k = -84mV$,<br>$v_{Ca} = 120mV$,$v_L = -60mV$, $v_1 = -1.2mV$,<br>$v_2 = 18mV$, $v_3 = 2mV$, $v_4 = 30mV$, $\phi = 0.08$
**Calcium dynamics**	
$g_{Ca} = g_{cabase}\frac{M_{pa}}{k_{pa}+M_{pa}}$<br>$\frac{d}{dt}Ca = k_{NMDA}I_{NMDA} - k_{Ca}I_{Ca} + \frac{Ca_0 - Ca}{\tau_{Ca}}$	$g_{cabase} = 5.3\ nS$, $k_{pa} = 0.01\ nM$<br>$Ca_0 = 500nM$, $k_{INMDA} = 10$, $k_{Ca} = 1$, $\tau_{Ca} = 10ms$
**AMPAR dynamics**	
$\frac{d}{dt}EPSP = I_{NMDA}/C$<br>$\frac{d}{dt}AMPAR = A_{MS}((a_{r1}(Ca - Ca_1)(A_m - AMPAR) -$<br>$a_{d1}AMPAR(Ca - Ca_1))S_{AMPA}) +$<br>$P_{1pa}^{ss}a_{remov}(\frac{I_{pre}}{1+I_{post}})(1 - AMPAR)$<br>$A_m = A_{MS}A_{mx}$<br>$A_{MS} = \frac{k_{am1}^{qq}}{P_{1pa}^{qq}}$<br>$\frac{d}{dt}S_{AMPA} = a_{rs}I_{post}(1 - S_{AMPA}) - a_{ds}S_{AMPA}$	$a_{r1} = 1e-7\ nM^{-1}ms^{-1}$, $A_m = 1e4$<br>$a_{d1} = 1e-5\ nM^{-1}ms^{-1}$<br>$Ca_1 = 509.5\ nM$, $A_{mx} = 2500$, $k_{am1} = 1.3\ nM$<br>$q_q = 2$, $s_s = 2$<br>$a_{rs} = 0.01\ pA^{-1}ms^{-1}$, $a_{ds} = 7e-4\ ms^{-1}$
