# Supplementary material for: Multi-scale modeling of the circadian modulation of learning and memory
Source: PLoS One. 2019 Jul 19;14(7):e0219915. doi: 10.1371/journal.pone.0219915 (PMC6641212; doi:10.1371/journal.pone.0219915)
Supplement: S4 Table — (PDF) [file pone.0219915.s004.pdf]

S4 Table. Day and night difference in degree of acquisition, and extinction

	LD			DL			DD		
# Test	Difference in Simulation	Difference in Experiment	Relative error	Difference in Simulation	Difference in Experiment	Relative error	Difference in Simulation	Difference in Experiment	Relative error
Acquisition									
1	47.4	20.5	1.3	47.4	5.5	7.6	13.2	34.5	0.6
2	59.8	45.5	0.3	60.0	27.0	1.2	16.9	44.0	0.6
3	62.7	30.5	1.0	62.8	30.0	1.0	18.0	26.0	0.3
4	63.1	15.5	3.0	63.2	31.0	1.0	18.3	16.5	0.1
5	63.0	25	1.5	63.2	41.0	0.5	18.3	6.5	1.8
6	62.9	43.5	0.4	63.0	38.5	0.6	18.3	21.0	0.1
Extinction									
1	0	0	0	0	0	0	0	0	0
2	0.21	0.249	0.5	0.21	0.21	0	0.26	0.30	0.14
3	0.36	0.49	0.2	0.36	0.31	0.15	0.39	0.50	0.2

Difference in Simulation = Normalized g_{AMPAR} at ZT3 - Normalized g_{AMPAR} at ZT15

Difference in Experiment = percent Freezing at ZT3 - percent Freezing at ZT15

Relative error = (| Difference in Simulation - Difference in Experiment |)/ Difference in Experiment
